# A SWI/SNF-specific Ig-like domain, SWIFT, is a transcription factor binding platform

**DOI:** 10.1101/2025.08.01.667725

**Published:** 2025-08-01

**Authors:** Siddhant U. Jain, Kaylyn E. Williamson, Alexander W. Ying, Ruidong Jerry Jiang, Aasha M. Turner, Kevin So, Maxwell P. Allison, Akshay Sankar, Daniel D. Sáme Guerra, Nazar Mashtalir, Henry W. Rohrs, Cheryl F. Lichti, Steven P. Gygi, Joao A. Paulo, Michael L. Gross, Cigall Kadoch

**Affiliations:** 1 Department of Pediatric Oncology, Dana-Farber Cancer Institute and Harvard Medical School, Boston, MA 02215, USA; 2 Broad Institute of MIT and Harvard, Cambridge, MA 02142, USA; 3 Department of Chemistry, Washington University in St. Louis, St. Louis, MO 63105, USA; 4 Departments of Pathology and Immunology, School of Medicine, Washington University in St. Louis, St. Louis, MO 63110; 5 Department of Cell Biology, Harvard Medical School, Boston, MA 02115, USA; 6 Howard Hughes Medical Institute, Chevy Chase, MD 20815, USA

## Abstract

Mammalian SWI/SNF (BAF) chromatin remodeling complexes modulate DNA accessibility and gene expression, however, the mechanisms by which these master regulatory complexes are targeted on chromatin remain incompletely understood. Here, we define SWIFT (SWI/SNF Ig-Fold for Transcription Factor Interactions) found on the SMARCD family of subunits within the core module as a transcription factor (TF) binding platform. We demonstrate that SWIFT is necessary and sufficient for direct interaction with the transactivation domain of a lineage-specific TF, PU.1, in vitro and in cells. A single amino acid mutation in SWIFT disrupts the PU.1-mSWI/SNF interaction, inhibits site-specific complex targeting and activity, and attenuates oncogenic gene expression and proliferation of PU.1-dependent AML cells. Dominant expression of SWIFT in isolation sequesters mSWI/SNF-interacting TFs and poisons TF-addicted cancer cells. Finally, we present SWIFT as an evolutionarily conserved domain that serves as a universal binding platform for diverse TFs, suggesting approaches for modulation of cell type and disease-specific transcription.

## Introduction

Timely and appropriate eukaryotic transcription requires highly coordinated, dynamic interactions among protein factors and DNA. Interplay between specialized proteins such as TFs, chromatin landscape features such as histone and DNA post-translational modifications, and regulatory machines such as chromatin remodeling complexes work in concert to control chromatin accessibility and binding of transcriptional apparati to target genes^1^. Specifically, the mammalian Switch/Sucrose Non-Fermenting (mSWI/SNF) family of chromatin remodeling complexes encompasses a heterogenous collection of 11–15 subunit protein entities that utilize the energy of ATP hydrolysis to alter DNA-nucleosome contacts and hence remodel chromatin architecture^2–7^. The mSWI/SNF complexes are found in three major configurations, termed canonical BAF (cBAF or BAF), polybromo-associated BAF (PBAF), and non-canonical BAF (ncBAF), each of which is demarcated by the incorporation of specific subunits and which exhibit different localization proclivities and nucleosome interactions on chromatin^8–16^. Within each complex, several families of two- or three- paralog subunits can be co-expressed and assembled into complexes in a mutually exclusive manner in the same cells, allowing for hundreds of combinatorial possibilities. Specialized mSWI/SNF assemblies play critical roles in development and cell type maintenance, such as ES cell identity, as well as in differentiation processes across a multitude of tissue types^17–24^. Studies examining the conditional deletion of mSWI/SNF subunit genes in vivo underscore the criticality of these factors for proper development and differentiation processes^25–29^. Further, genes encoding mSWI/SNF complexes are mutated in over 20% of human cancers, making these remodelers the most frequently perturbed cellular entities second only to TP53^3,30,31^.

Canonical BAF (cBAF) complexes exhibit a primarily TSS-distal localization pattern on chromatin, with occupancy most strongly enriched at enhancers and super-enhancers at which lineage-specific transcription factors (TFs) target and help orchestrate proper gene expression^8,14,15,32–34^. Comprehensive studies such as genome-scale CRISPR-based fitness screens reveal that mSWI/SNF complex genes either show strong co-dependencies with TFs, indicating their shared role in supporting oncogenesis, or, conversely, that mSWI/SNF subunit mutations inhibit proper DNA accessibility generation over lineage-specific enhancers required for proper differentiation^35–38^. Taken together, these findings implicate mSWI/SNF complexes, and specifically, cBAF complexes, as a critical determinants of lineage-specific, TF-mediated gene expression.

Importantly, the subunits comprising the mSWI/SNF family of remodeling complexes do not contain sequence-specific DNA-binding domains, and contain a limited set of histone reader domains, leaving open the question as to how mSWI/SNF complexes are differentially targeted to specialized sites genome-wide in cells. While we have recently determined the features of the histone landscape that impact mSWI/SNF family complex binding and nucleosome remodeling activities, these findings are insufficient in isolation to explain the highly distinct mSWI/sNF complex targeting profiles observed across diverse human cell and tissue types. Several studies to date have highlighted indirect protein-level interactions between TFs and mSWI/SNF complexes, particularly in cancer contexts in which TFs are overexpressed or exist as part of fusion proteins^39–41^. Several reports also suggest that a wide range of sequence-specific TFs interact with mSWI/SNF complexes during the course of normal cellular development and differentiation and depend on MSWI/SNF complexes to achieve- cell type specific accessibility, specifically at distal enhancer regions^21–23,33,42–50^. However, to date there exists no understanding as to whether these interactions are direct nor have the subunits or region(s) that mediate potential TF interactions with mSWI/SNF multi-component complexes been identified. Indeed, defining the mechanisms by which TFs spanning different families and functional groups guide the targeting and genome-wide activities of this major class of chromatin remodeling complexes is critical to understanding developmental and differentiation processes, the biochemical underpinnings of TF-addicted cancers, and may inform new strategies for therapeutic intervention.

## Results

### Transcription factor expression and cognate motif density directs tissue-specific genome-wide mSWI/SNF complex localization

**Figure 1.**
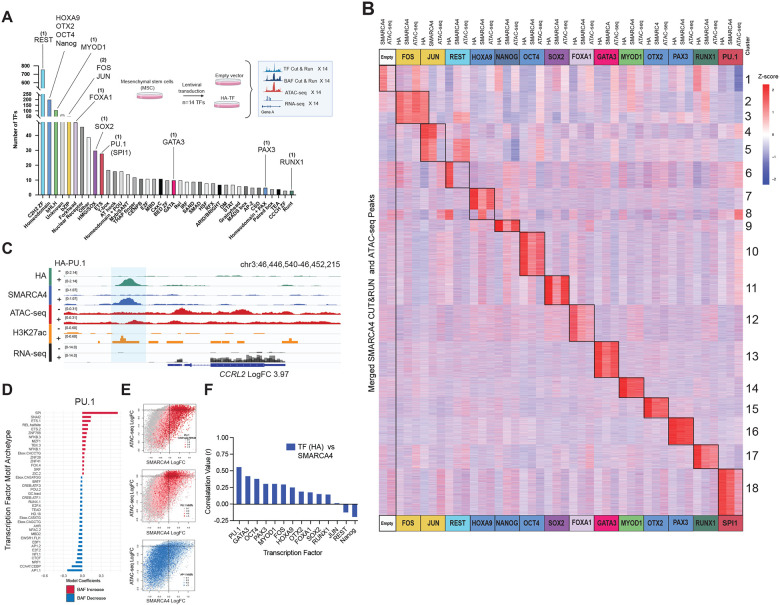
Transcription factors direct mSWI/SNF complex targeting and activity genome-wide. **A.** Bar graph depicting human transcription factors grouped by family; those selected for HA-tagged expression and genomic studies in hMSCs are indicated. **B.** Heatmap displaying the z-score normalized RPKM occupancies of HA-tagged TFs (as shown on top in colored boxes), SMARCA4, and DNA accessibility (ATAC-Seq) at all merged SMARCA4 and ATAC-Seq sites across all samples. Z-scores were calculated for each experiment individually prior to grouping of columns by cell lines for data visualization. Unguided hierarchical clustering was performed on using z-score normalized values for HA-TF CUT&RUN, which identified 18 distinct clusters as shown on the right. **C.** Representative site showing TF-dependent mSWI/SNF localization and accessibility at the *CCRL2* locus. RPKM-normalized enrichment of HA-tagged PU.1, SMARCA4, H3K27ac, DNA accessibility (ATAC-Seq) and gene expression (RNA-Seq) are shown. **D.** GLMnet motif enrichment analysis was performed to identify top TF motifs underlying SMARCA4 peaks that displaying gain of SMARCA4 enrichment (in red) and loss of enrichment (in blue) upon TF overexpression. **E.** Scatterplots displaying the correlation between change in SMARCA4 occupancy (x-axis) and DNA accessibility measured by ATAC-Seq (Y-axis) in MSCs expressing PU.1 compared to empty vector. Color key indicates PU.1 RPKM enrichment (top panel), number of PU.1-motifs (middle panel) and number of AP.1 motifs (lower panel). **F.** Bar chart displaying Pearson correlation coefficients (r) for the correlation between changes in SMARCA4 occupancies and HA-tagged transcription factor occupancies in hMSC cell lines expressing the TF indicated on X-axis. PU.1 and GATA3 were top-ranked TFs for their ability to guide SMARCA4 (mSWI/SNF complexes) to de novo TF motif-enriched target sites on chromatin.

Genome-wide chromatin occupancy of mSWI/SNF complexes, specifically, cBAF complexes, is influenced by the expression of lineage-specific transcription factors (TFs). Indeed, tissue and cell type-specific mSWI/SNF complex sites, largely found at distal enhancers, are characterized by the enrichment of DNA motifs corresponding to one or more key lineage TFs (Fig S1A–G). Generally, mSWI/SNF complex occupancy over sites enriched with lineage-specific TFs runs as a function of TF mRNA expression in a given tissue type, with site-specific occupancy localized near TF target genes (Fig. S1H–L). There are over 30 different families of human transcription factors (TFs) as characterized by the structure of their DNA binding domains^51^. To examine the impact of TF expression on mSWI/SNF complex occupancy and resulting accessibility, we individually expressed a diverse collection (n=14) of hemagglutinin (HA) N-terminally tagged TFs spanning 10 TF families in an isogenic setting, human adipose-derived mesenchymal stem cells (MSCs) and profiled resulting TF and mSWI/SNF complex occupancy (CUT&RUN), chromatin accessibility (ATAC-seq) and transcriptional activity (RNA-seq) (Fig. 1A, S2A–B). MSCs were chosen for these studies because of their relative epigenetic plasticity and their capacity to differentiate into a wide range of mesodermal lineages such as adipose, bone, and muscle tissues in response to overexpression of lineage-specific TFs^52^.

Intriguingly, overexpression of several TFs, including PU.1, GATA3, MYOD1, OTX2, and PAX3 in MSCs lead to de novo gains in mSWI/SNF complex occupancy and DNA accessibility as measured by SMARCA4 CUT&RUN and ATAC-seq peak signals, respectively, at previously mSWI/SNF-deficient and inaccessible genomic loci (Fig. 1B, S2C). Notably, overexpression of 14 specific TFs, spanning 10 distinct TF families resulted in highly specific de novo targeting of mSWI/SNF complexes on chromatin to previously inaccessible distal enhancer sites that undergo chromatin remodeling as a result (Fig. 1C). Strikingly, each TF results in MSWI/SNF complex targeting to a very specific set of genomic sites, while few sites are targeted by more than one TF, suggesting TF-specific mSWI/SNF guidance. With the exception of REST, a factor implicated in both transcriptional activation and repression through its association with various chromatin modifying complexes such as coREST, G9a, and components of the mSWI/SNF complex^53^, TF overexpression in hMSCs resulted in highly specific sets of sites of mSWI/SNF targeting genome-wide that are characterized by strong enriched in the motif of the overexpressed factor (Fig. 1C). Nearest gene expression of de novo complex target sites was significantly increased in the setting of TF expression (Fig. S2D). These results are exemplified at the *CCRL2, MECOM*, *KCNK2*, and loci for PU.1, GATA3, and PAX3, respectively (Fig. 1C, S2E–F).

Expression of TFs resulted in skewing of mSWI/SNF occupancy to sites highly enriched for the archetypal motif of the primary overexpressed TF (as is shown for PU.1 and GATA3) coupled with concomitant depletion of occupancy over sites of mSWI/SNF targeting in unmodified (empty vector) MSCs, such as AP.1 family archetype motifs, as visualized by an unbiased linear regression model (GLMnet) of the logFC in SMARCA4 occupancy as a function of TF motif archetype enrichment under SMARCA4 target sites (Fig. 1D, S2H–I). This supports a potential model in which tissue-specific TFs dominate, either by expression (abundance), affinity, or a combination thereof, over general mSWI/SNF-interacting TFs such as AP-1, FOS, JUN to direct mSWI/SNF complex localization genome-wide. Notably, sites of greatest mSWI/SNF-complex occupancy gains and increases in underlying accessibility were sites enriched with the greatest number of motifs corresponding to the overexpressed TF (Fig. 1E, S2J). These sites are also those most highly bound by the expressed TF itself, suggesting motif-based concentration dependent recruitment of the TF and mSWI/SNF complexes to de novo sites (Fig. 1E, Fig. S2H,J). This is demonstrated at the single locus level at distal enhancer sites with 5, 3, and 1 PU.1 (PU.1) archetypal motifs nearest the *A2M, HGD*, and *CD48* loci respectively, to which PU.1 itself, as well as mSWI/SNF complexes are recruited in a motif-dependent manner resulting in a gradient of changes in accessibility and nearest gene expression (Fig. S2J). Sites with AP-1 family TF consensus motifs were increasingly depleted for mSWI/SNF complex occupancy and chromatin accessibility upon expression of lineage-specific TFs, further corroborating the ‘skewing’ away from these sites observed in the unbiased linear regression model (Fig. 1E, S2H). Taken together, these results indicate a TF-specific, TF archetype motif concentration-dependent impact on the genome-wide occupancy of mSWI/SNF chromatin remodeler complexes and their activities.

### Mass-spectrometry-based footprinting resolves a PU.1-mSWI/SNF core module interaction region

**Figure 2.**
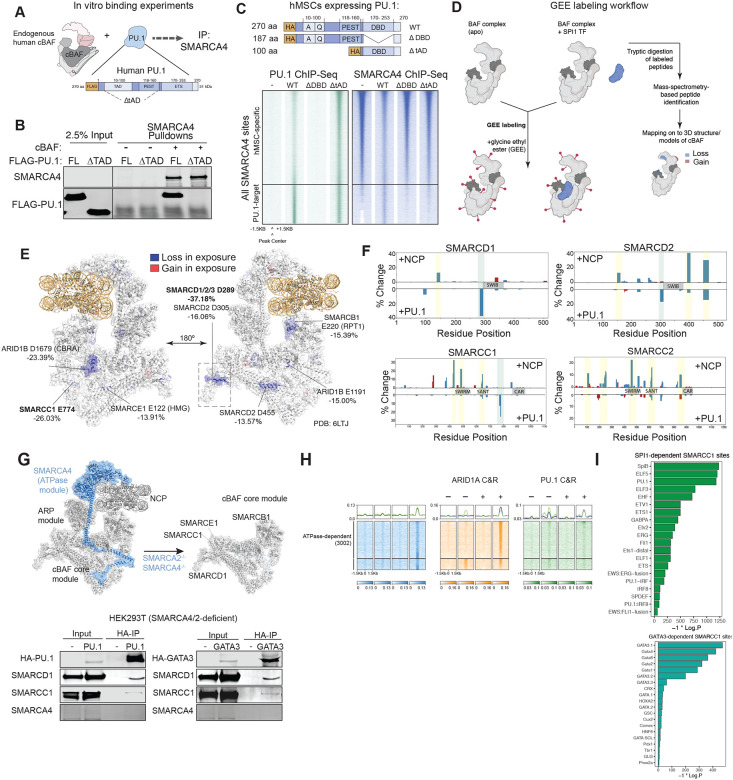
PU.1 interacts with the mSWI/SNF core structural module. **A.** Schematic for in vitro incubation experiments using endogenous, fully-assembled human cBAF complexes and full-length human PU.1. Domains within PU.1 are indicated. **B.** SMARCA4 pulldown experiments performed with 2.5 μg of purified cBAF incubated with 10-fold molar excess of PU.1 wild-type or PU.1 deltAD (delaa1-160). **C.** (Top) schematic for HA-tagged PU.1 variants introduced into human MSCs for PU.1 and SMARCA4 ChIP-seq experiments; (Bottom) heatmaps depicting PU.1 and SMARCA4 occupancy across all merged SMARCA4 sites in empty vector, and PU.1 WT, delDBD, and delTAD variant conditions. PU.1-specific target sites are indicated. **D.** Schematic for mass-spec-based GEE protein footprinting experiments performed with endogenous human cBAF complexes and full-length PU.1. E. cBAF peptides displaying GEE-labeling changes were mapped onto the 3D structure of NCP-bound cBAF, with SMARCD1 modeled with AlphaFold2 and superimposed on PDB:6LTJ. F. Bar charts across mSWI/SNF SMARCD and SMARCC subunits depicting %GEE labeling change upon incubation with NCP (top) or PU.1 (bottom). **G.** (Top), schematic depicting mSWI/SNF complexes with a stable and independently assembled core upon removal of the ATPase subunits; (bottom) Expression of HA-tagged PU.1 or GATA3 TFs in SMARCA4/SMARCA2 dual-deficient HEK-293T cells results in mSWI/SNF complex interactions. **H.** Heatmaps depicting occupancy of mSWI/SNF complexes (SMARCC1, SMARCA4) with and without expression of HA-tagged TFs PU.1 and GATA3. **I.** HOMER motif analyses performed on SMARCA4 sites gained in the setting of TF overexpression.

Based on our findings that the PU.1 (and GATA3) TFs can relocalize mSWI/SNF occupancy and activity genome-wide, we next sought to determine whether they directly interact. We purified human PU.1 and cBAF complexes in mammalian cells (HEK-293T) using overexpression and subsequent density sedimentation studies to ensure protein purity and isolation (Fig. 2A, S3A–B)^9,11^. Indeed, PU.1 interacts directly with cBAF complexes in the absence of any additional factors (Fig. 2B). Notably, deletion of the PU.1 transactivation domain (tAD, residues 10–160) nearly completely abrogated this interaction (Fig. 2B). In line with these in vitro results, overexpression of a PU.1 ΔtAD deletion mutant in hMSCs failed to recruit mSWI/SNF complexes, marked by SMARCA4, to PU.1 target sites, relative to full-length PU.1, suggesting that the tAD region is necessary both for direct biochemical interaction and PU.1-mediated mSWI/SNF targeting (Fig. 2C).

Next, we developed and implemented a biophysical approach to define the cBAF complex subunit or interface with which the PU.1 TF interacts. We used a chemical labeling method using water-soluble carbodiimde in combination with glycine ethyl ether (GEE) to specifically modify solvent-accessible carboxyl groups on glutamate (E) and aspartate (D) residues^54–56^, thus reporting on changes in surface exposure upon PU.1-cBAF interaction in vitro (Fig. 2D). In parallel, we performed these studies with cBAF incubated with recombinant nucleosomes, a well-documented and structurally resolved interaction, as a control using the same conditions. Notably, we identified 75 unique changes in GEE labeling upon PU.1 binding, including 57 sites with decreased and 18 sites with increased surface exposure (Fig. S3C–D). GEE labeling changes detected on cBAF complexes upon NCP binding mapped to the helicase domain of the SMARCA2/SMARCA4 ATPase subunits and other structural core subunits that have been shown to undergo configurational rearrangement upon NCP engagement (Fig. S3C,E)^9,57–59^. Importantly, the top 7 sites with the most substantial reductions in surface exposure upon PU.1-cBAF incubation all mapped to subunits of the initial mSWI/SNF structural core module including SMARCD and SMARCC subunits (Fig. 2E–F, S3D). Residue D289 of the SMARCD1 and SMARCD2 subunits (shared between paralogs) exhibited the largest reduction in surface accessibility upon PU.1 incubation (−37.18% decrease relative to unbound). These data inform potential sites of direct interaction of the PU.1 TF with the cBAF complex core module and nominate the SMARCD1/2/3 subunits as those containing regions with putative direct interfaces.

We have previously demonstrated that a structural core module of cBAF complexes comprising of SMARCD1/2/3, SMARCC1/2, SMARCB1, SMARCE1 and ARID1/ARID2 subunits is formed in cells independent of the presence of the catalytic ATPase module containing SMARCA2/4 (Fig. 2G) subunits^9,60^. Consistently, PU.1 and another lineage-specific TF, GATA3, both co-immunoprecipitated SMARCC1 and SMARCD1 subunits in HEK-293T cells genetically deficient in SMARCA2/4 subunits, at comparable levels as in wild-type (WT) HEK-293T cells (Fig. 2G, S4A). These results suggest that cBAF core is sufficient to interact with PU.1 and GATA3 TFs in cells. Further, in a cancer cell line defined by deficiency in both SMARCA4 and SMARCA2 expression and hence lacking the entire ATPase module (BIN-67 SCCOHT cells^60–63^, we found that expression of PU.1 or GATA3 resulted in substantial redirection of core module occupancy on chromatin (Fig. 2H, S4B–E), with TF-specific de novo mSWI/SNF sites showing corresponding motif enrichment (Fig. 2I). As expected, reintroduction of WT but not ATPase-dead (K785R) SMARCA4 into BIN-67 ATPase-deficient cells resulted in substantial increases in DNA accessibility^60^ and additional mSWI/SNF target sites, including those targeted by PU.1 and GATA3 TFs (Fig. S4F–I).

### An Ig-like fold, SWIFT, in the mSWI/SNF structural core interacts with PU.1 in vitro

**Figure 3.**
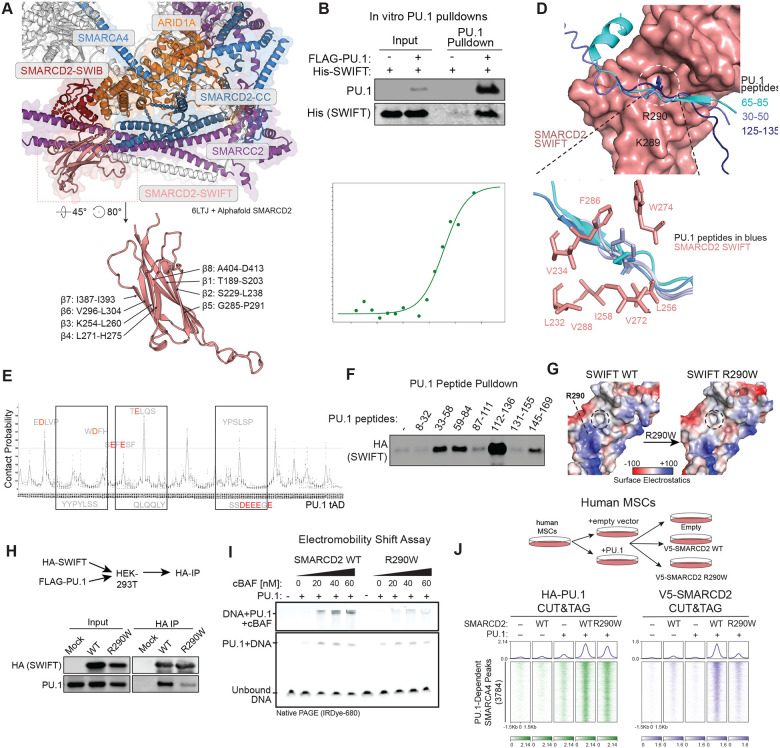
The SMARCD2 SWIFT domain is necessary and sufficient to for interaction with PU.1 in vitro and in cells. **A.** (Top) mSWI/SNF core subunits showing interactions between SMARCD2 (salmon) coiled coil domain with SMARCC2 (blue), and between SWIB domain with ARID1A (orange); (Bottom) Structure of the SMARCD2 Ig-like fold, SWIFT; PDB: 6LTJ+ Alphafold SMARCD2. **B.** Immunoblots of FLAG-PU.1 pulldown experiments performed by incubation of 0.5 μM FLAG-tagged PU.1 was incubated with 2 μM SMARCD2 SWIFT domain. **C.** Kd model fit for interaction between SMARCD2 SWIFT domain and PU.1 using microscale thermophoresis (Kd = 3.6uM). **D.** AlphaFold3 multimer modeling displaying the putative interaction between PU.1 20-mer peptides and a hydrophobic pocket on the surface of SMARCD2 SWIFT domain. **E.** Contact probability scores of interaction between each residue on the transactivation domain of PU.1 with the residues within the hydrophobic pocket on SWIFT domain determined by Alphafold modeling of all consecutive PU.1 10-mer peptides. Line shows the mean of all outcomes for a given residue. **F.** Immunoblots of eluates from peptide pulldown of 7 PU.1 peptides (25-mer) spanning full transactivation (tAD) domain. **G.** 3D-surface rendering of surface electrostatic potential of wildtype SMARCD2 SWIFT and R290W point mutation near the hydrophobic pocket (shown in black dashed circle). H. Immunoblots displaying the elutates from Immunoprecipitation of HA-tagged SWIFT domain from HEK-293T cells expressing HA-tagged SWIFT wildtype or R290W mutant and FLAG-tagged PU.1. **I.** Electromobility shift assays (EMSA) performed on complex formed by dsDNA containing a PU.1 binding motif and PU.1 incubated with cBAF complexes containing wildtype SMARCD2 or R290W mutation. Upper panel shows the supershift caused by addition of cBAF complexes. **J.** Heatmaps depicting PU.1 and V5-tagged SMARCD2 occupancy (CUT&RUN) in hMSCs expressing empty vector, V5-tagged WT SMARCD2 or SMARCD2 R290W mutant in the presence or absence of PU.1.

GEE-labeling experiments coupled with our results demonstrating that TFs including both PU.1 and GATA3, are able to bind to and direct the mSWI/SNF core module genome-wide motivated us to identify a potential direct engagement interface. The region on cBAF complexes that showed highest reduction in GEE labeling upon PU.1 incubation mapped to a functionally uncharacterized domain in the SMARCD subunits (Fig. 2). Encompassing the residues that displayed the greatest changes in surface exposure is a ~26 KDa domain that contains a sandwich arrangement of 8 anti-parallel β-strands, characteristic of the immunoglobulin folds (Ig-fold) (Fig. 3A, S5A–B)^64,65^. However, intriguingly, the SMARCD1/2 β-sandwich deviates from conventional Ig-folds by incorporating a SWIB domain within loop 7, which we and others have demonstrated interacts with the AIRD1A/1B or ARID2 subunits and is likely necessary for complex assembly (Fig. 3A, S5A)^9,58,66^. We performed primary sequence alignment which suggested that the SMARCD1/2 Ig-fold is distantly related to the YEATS domains found in chromatin modifiers (AF9, ENL etc.) (Fig. S5C)^67–70^. However, aromatic residues within loop 6 of the YEATS domain of AF9, which constitute an aromatic tunnel and are essential for binding histone acetylation/crotonylation through ∏-∏-∏ stacking, are notably absent from the SMARCD1/2/3 Ig-fold, suggesting a divergent function (Fig. 3A, S5C). Based on our characterization of this SMARCD Ig-fold, we will henceforth refer to it as **SW**I/SNF **I**g-**F**old for **T**F-interactions (SWIFT).

**Figure 4.**
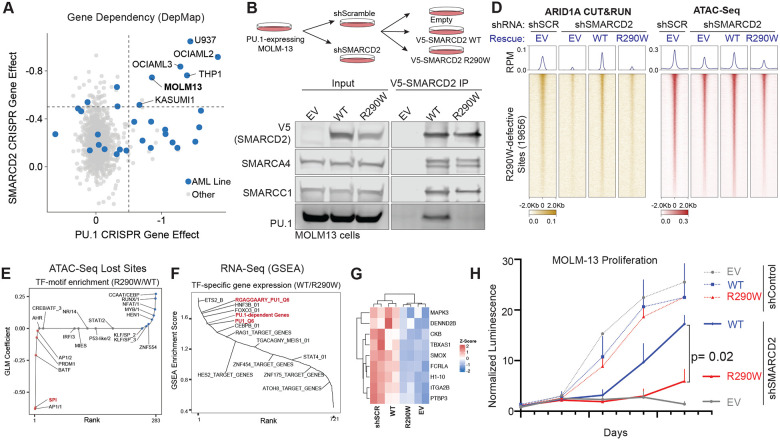
SMARCD2 SWIFT is necessary to sustain PU.1-dependent transcription and cancer cell proliferation. **A.** Scatterplot displaying CRISPR gene effect scores (dependency) for SMARCD2 (Y-axis) and PU.1 (X-axis) across n=900 cancer cell lines. Acute myeloid leukemia (AML) cell lines are indicated in blue. **B.** Schematic displaying the experimental strategy to identify the function of PU.1-SWIFT interaction interface in MOLM-13 AML cells. Endogenous SMARCD2 is suppressed using shRNA followed by exogenous expression of shRNA-resistant SMARCD2 WT or R290W mutant variants. **C.** Immunoblots performed on inputs and V5 immunoprecipitations of nuclear extracts isolated from MOLM-13 cells containing either WT or R290W mutant SMARCD2. **D.** Heatmaps depicting ARID1A chromatin occupancy (CUT&RUN) and DNA accessibility (ATAC-seq) at cBAF-occupied sites in MOLM-13 cells rescued with empty vector (EV), WT SMARCD2, or R290W mutant following shRNA-mediated endogenous SMARCD2 suppression. **E.** Motif enrichment analysis at genomic sites with reduced chromatin accessibility in cells rescued with the SMARCD2 R290W mutant. **F.** Gene set enrichment analysis of genes downregulated in cells rescued with SMARCD2 R290W mutant compared to SMARCD2 WT. PU.1 target gene sets are highlighted in red. **G.** Z-score normalized RNA-Seq expression of PU.1-target genes in MOLM-13 AML cells in control (shScramble) or shSMARCD2 rescued with either EV, WT SMARCD2 or R290W SMARCD2^94^. **H.** Cell proliferation (normalized luminescence) of MOLM13 cells with SMARCD2 knockdown (or a scrambled control) rescued with shRNA-resistant SMARCD2 WT or R290W transgenes. Proliferation was monitored for 12 days following infection and selection (data points represent 2 independent biologic replicates, each with 2 technical replicates).

We next purified the SMARCD2 YEATS-like SWIFT domain and full-length PU.1 from E.coli and mammalian Expi293TF cells, respectively, and incubated these in vitro (Fig. S5D). Remarkably, SMARCD2 SWIFT displayed direct interaction with purified PU.1 outside of the context of fully assembled mSWI/SNF complexes in in vitro pulldown experiments (Fig. 4B). Using microscale thermophoresis (MST), we determined the dissociation constant for the interaction between the SMARCD2 SWIFT domain and PU.1, as 3.6 μM (Fig. 3C), which is comparable to those measured for TF-P300 interactions and meaningful protein-protein interactions^70–73^. These data establish that the SMARCD2 SWIFT domain is sufficient for interaction with PU.1 *in vitro*.

Next, we modeled the SMARCD2 SWIFT and PU.1 interaction using Alphafold 3, which determined interaction between PU.1 residue L73 within its tAD domain with a hydrophobic pocket formed between the two sandwiched β-sheets of the SWIFT domain (F286, W274, V288, I272) with an aggregate contact probability of 81% and lowest predicted alignment error of 4.6 (Fig. 3D). TF tADs are not conserved evolutionarily or between families; however, these intrinsically disordered regions contain Short Linear Motifs (SLiMs) that are universally enriched in hydrophobic and acidic residues and possess an overall negative charge^71,74–77^. These features led us to hypothesize that the aliphatic and aromatic residues in the PU.1 tAD make polyvalent interactions with the SWIFT hydrophobic cage (Fig. S5E). We next performed in silico interaction prediction between the SWIFT domain and all consecutive 10-mer peptides in the PU.1 tAD and identified multiple putative PU.1 residues engage with the same hydrophobic pocket on SWIFT (Fig. 3E). We identified acidic (Asp/Glu) or electronegative residues adjacent to nearly all residues within the hydrophobic pocket. Intriguingly, the surface adjacent the SWIFT hydrophobic pocket contains several solvent-exposed positively charged residues, in particular, K289 and R290 (Fig. 3D, 3G, **left**). We thus hypothesized that the PU.1 tAD makes polyvalent interactions with SWIFT for which aliphatic and aromatic residues on the tAD engage with the hydrophobic pocket and that the adjacent amino acids make electrostatic or hydrogen bonds with the positively charged surface on SWIFT.

Based on these in silico predictions, we synthesized seven 25-aa long peptides spanning the entire PU.1 tAD domain and tested their ability to bind SWIFT domain (Fig. 3E–F). We found that 4 of the 7 peptides bound SMARCD2 SWIFT domain with varying levels, suggesting that the PU.1 tAD contains multiple polyvalent sites able to engage with SWIFT (Fig. 3E–F). Strikingly, the peptide that showed the highest ability to bind SWIFT domain contained a stretch of acidic and electronegative residues (Q128-E137) near L124, suggesting that the positively charged surface on SWIFT contributes to TF binding affinity.

We next integrated human cancer-associated mutations (COSMIC) and predictions to identify single-residue point mutations within the SWIFT domain, which could impair its interaction with PU.1. Among these, a SMARCD2 R290W mutation mapped to the residue next to the SWIFT hydrophobic pocket predicted to interact with PU.1 tAD (Fig. 3G, S5E). Supporting the in silico predicted interaction model, R290W point mutation, which reduces the positive surface charge on SWIFT, markedly reduced its interaction with PU.1 in cells (Fig. 3G–H).

Finally, to characterize the PU.1-mSWI/SNF interaction through SWIFT, we purified fully assembled mSWI/SNF complexes containing either WT or R290W mutant variant SMARCD2 (Fig. S6A). As expected from the solvent-exposed R290 side chain within the SWIFT domain structure, the mutation of the R290 residue within SMARCD2 did not affect mSWI/SNF complex integrity or remodeling activities (Fig. S6A–F). However, consistent with impaired interaction with PU.1, mSWI/SNF complexes containing SMARCD2 R290W failed to supershift the PU.1-DNA complex in vitro (Fig. 3I). Consistent with our *in vitro* results, expression of PU.1 in hMSCs recruited V5-tagged wild-type SMARCD2 to PU.1 target sites but displayed markedly impaired recruitment of SMARCD2 R290W mutant-containing complexes (Fig. 3J). Taken together, our results demonstrate that the SWIFT Ig-like domain is necessary and sufficient for the mSWI/SNF interaction with PU.1 in vitro and in cells.

### A single-residue SWIFT domain mutation blocks PU.1-mediated mSWI/SNF targeting and impairs oncogenic gene expression and proliferation in transcription factor-driven AML cells

**Figure 5.**
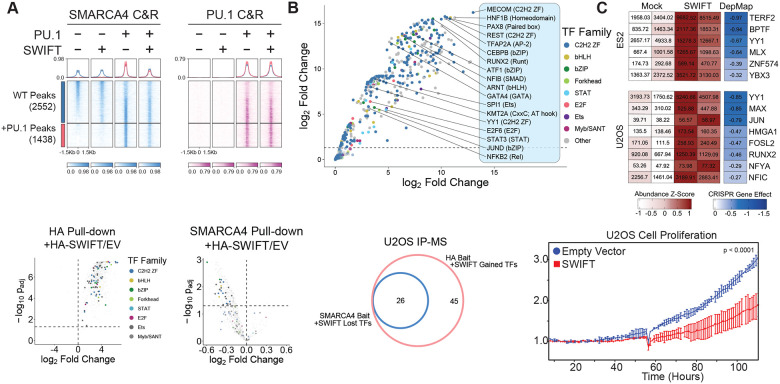
SWIFT is an evolutionarily SWI/SNF-specific platform for diverse transcription factor interactions. **A.** Heatmap displaying the CUT&RUN-determined occupancy of SMARCA4 and PU.1 in hMSCs expressing PU.1 in the presence or absence of SMARCD2 SWIFT domain expression. **B.** Scatterplot displaying the composite enrichment of transcription factors bound by 200 nM SMARCD2 SWIFT (versus mock control) incubated in nuclear extracts isolated from U2OS osteosarcoma, ES2 and OVISE ovarian carcinoma, and NCI-H1048 small cell lung cancer cell lines as detected by mass spectrometry (n= 2 replicates for each cell line). **C.** Heatmaps displaying the enrichment of TFs bound by SMARCD2 SWIFT from ES2 and U2OS cell nuclear extracts (mock shown as control) and their CRISPR gene dependency scores (DepMap). **D.** Scatter plots showing enrichment of TFs, colored by family, in SWIFT domain pulldown (left) and SMARCA4 pulldown (right) in the setting of HA-SWIFT overexpression in U2OS cells. Selected TFs are labeled. **E.** Venn diagram depicting overlap between TFs enriched (gained) in SMARCD2 SWIFT domain IP-MS and those reduced in mSWI/SNF (SMARCA4) binding. **F.** Proliferation of U2OS cells overexpressing SWIFT domains, monitored by eSight. P-value p<0.0001 of difference in the slope of exponential growth for each line (n= 3 replicates) is indicated.

We next sought to determine whether alteration of the SMARCD2 SWIFT domain-PU.1 interaction is necessary to support the chromatin occupancy, gene regulatory and proliferative signatures of human cancers dependent on the PU.1 transcription factor as a way to probe the concept that TF interactions with this domain may be required for transcription factor-’addicted’ cancers. To this end, PU.1 is central in orchestrating lineage commitment in the myeloid and macrophage lineage and is a top-ranked vulnerability in AML cell lines where it functions to maintain myeloid progenitor expression programs and self-renewal^28,78–84^ (Fig. S7A). Intriguingly, integrating results from genome-scale CRISPR-Cas9-based fitness screens performed across >1000 cancer cell lines, we find that AML cell lines exhibit among the highest co-dependency scores on both PU.1 and SMARCD2, suggesting the possibility that the interaction between PU.1 and SMARCD2 is necessary for oncogenic transcriptional functions in these cells (Fig. 4A, S7A). To assess this, we suppressed endogenous expression of SMARCD2 in MOLM13 cells (using shRNA) with simultaneous rescue of either WT SMARCD2 or SWIFT domain binding-deficient R290W mutant SMARCD2 using lentiviral overexpression (Fig. 4B). Consistent with our in vitro biochemical results and in-cell results, complexes containing the SMARCD2 R290W nearly completely ablated their interaction with PU.1, while maintaining interactions with other mSWI/SNF subunits (Fig. 4C, S7B). We next aimed to define the impact of the SMARCD2 SWIFT domain mutation on the targeting and accessibility generation of mSWI/SNF complexes in human AML cells using CUT&RUN and ATAC-seq, respectively. As expected, loss of SMARCD2 resulted in markedly decreased mSWI/SNF chromatin occupancy and accessibility genome-wide (Fig. S7C–D). Importantly, genome-wide, this loss was restored roughly equivalently by the WT and R290W SMARCD2 variants (Fig. S7E–F). Intersecting the restored sites achieved by WT SMARCD2 and R290W SMARCD2 identified a collection of 19,656 sites that required WT SMARCD2 for mSWI/SNF targeting in AML cells (Fig. 4D, S7F). Over these sites, targeting of mSWI/SNF complexes marked by both ARID1A and SMARCA4, as well as accessibility was markedly decreased (Fig. 4D, S7F–G). Distance-to-TSS analyses revealed these sites largely over distal enhancers (Fig S7H); motif analysis performed on these sites identified PU.1 motif as the top motif affected, with shuffling of chromatin occupancy and accessibility across motifs genome-wide (Fig. 5E). PU.1 occupancy was also reduced over these sites in the SMARCD2 KD and SMARCD2 R290W conditions, likely owing to reduced accessibility and open motif availability for binding (Fig. S7G). Finally, we examined the impact of the R290W mutant on MOLM-13 AML gene expression using RNA-seq. Notably, GSEA analyses performed in SMARCD2 WT and mutant rescue conditions revealed PU.1 target genes among top-scoring gene sets (Fig. 5F–G, S7I). Key target genes important in the maintenance of AML leukemogenesis were rescued in expression following SMARCD2 knockdown only by WT SMARCD2 and not R290W mutant SMARCD2 (Fig. S7I). Finally, as MOLM-13 cells are dependent on PU.1 for proliferative maintenance (Fig. 5A, S7A), we sought to determine the impact of SMARCD2 loss and the ability of either WT SMARCD2 or mutant R290W SMARCD2 to rescue cancer cell line proliferation in culture. Strikingly, knockdown of SMARCD2 attenuated proliferation of MOLM-13 cells; rescue of this proliferative defect was achieved only with SMARCD2 WT and not with the R290W mutant (Fig. 5H). Together, these data substantiate our biochemical results by demonstrating that the SMARCD2 SWIFT domain is necessary for recruitment of mSWI/SNF by PU.1 in cells and the subsequent activity at PU.1 target genes necessary to uphold cancer cell proliferation.

### SWIFT is a mSWI/SNF-specific evolutionarily conserved transcription factor binding hub

Our experiments thus far revealed that SWIFT is necessary and sufficient for interaction with the PU.1 transcription factor in vitro and in cells. We next expressed PU.1 in hMSCs along with the SMARCD2 SWIFT domain (SMARCD2 aa185–312) that is sufficient for interaction with PU.1 but cannot assemble into mSWI/SNF complexes due to the absence of its SWIB domain and N- and C-terminal helices necessary for interaction with core subunits^9,57–59,66^. Notably, while ectopic expression of PU.1 led to substantial de novo targeting of SMARCA4 to PU.1-target sites genome-wide, expression of PU.1 with concomitant expression of the SMARCD2 SWIFT domain markedly reduced SMARCA4 occupancy at these sites (Fig. 5A, S8A–B). Together, these data indicate that expression of the PU.1-interacting SWIFT domain in isolation blocks recruitment of endogenous mSWI/SNF at PU.1 target sites by dominantly interacting with, or “sequestering”, PU.1.

Evolutionary analyses of primary sequence of SWIFT domain and structural conservation shows that SWIFT is a SWI/SNF-specific Ig-fold, evolved in the earliest SWI/SNF complexes in yeast and is maintained throughout the phylogeny (Fig. S8C). This YEATS-like domain is substantially distinct from those of other YEATS-containing proteins such as ENL, AF9, and YEATS2, which have been shown to engage with histone tails^67,69,70^. Notably, in the yeast SMARCD2 homolog, SNF12, the YEATS domain-specific aromatic residues that are necessary for interaction with histone H3 acylation are missing, suggesting that this newly characterized SWIFT domain evolved separately from the distantly related YEATS domain. This evolutionary conservation of the SWIFT domain led us to define whether this is TF-binding platform that can accommodate a wide range of mammalian TF families to enable SWI/SNF complex recruitment. Of note, while the primary sequences of TF tAD domains are not conserved between and among TFs of different species, the hydrophobicity and overall negative charge is indeed a common characteristic of tADs across species.

To test whether SWIFT interacts with a wide number of TFs, we purified FLAG- and HA-tagged SMARCD2 SWIFT domains and incubated these with nuclear extracts from five distinct cell types (cancer cell lines) isolated by ammonium sulfate extraction, followed by HA-immunoprecipitation and mass spectrometry (MS) (Fig. 5B, S8D–E). Intriguingly, we detected significant enrichment of several TFs that specifically co-immunoprecipitated with the SMARCD2 SWIFT domain including YY1, JUN, FOSL2, and RUNX2, relative to mock control (Fig. 5C, S8E). These data suggest that other transcription factors also physically interact with mSWI/SNF complexes through the SMARCD SWIFT domain, hence our characterization and naming of the as-of-yet unnamed domain of unknown function as the SWI/SNF immunoglobulin fold for transcription factors. Notably, several SWIFT domain-interacting TFs represented top dependencies in the cancer cell lines in which interactions were identified such as BPTF and TERF2vin ES cells and YY1 and MAX in U2OS sarcoma cells (Fig. 5C). Further, we expressed SWIFT in U2OS cells and performed IP-MS studies to identify factors enriched on HA-SWIFT and simultaneously decreased in abundance on mSWI/SNF complexes via SMARCA4 IP. Notably, TFs such as RUNX1, RUNX2, TP53 among others, engaged with the dominantly expressed HA-SMARCD2 SWIFT domain and showed reduced engagement with SWIFT domain-containing mSWI/SNF complexes (Fig. 5D–E).

Given these results, we then speculated that ectopic expression of the SMARCD SWIFT domain in isolation could “poison” the required binding platform for TFs upholding cancer cell proliferation by blocking the interaction of essential TFs with endogenous mSWI/SNF complexes. Indeed, overexpression of the SMARCD2 SWIFT domain in U2OS osteosarcoma and ES2 ovarian cancer cell lines significantly attenuated proliferation(Fig. 5F, S8F). Together, these data highlight that exogenously expressed SWIFT dominantly blocks endogenous mSWI/SNF complexes from interacting with TFs, resulting in failure of TF-mediated mSWI/SNF targeting and oncogenic proliferation.

## Discussion

The mSWI/SNF core is the first to assemble in mSWI/SNF formation^11^, and lacks DNA sequence specific DNA-binding domains, leading to the hypothesis that direct interaction-based mechanisms beyond histone reader functions are likely to guide divergent complex localization across cells and tissue types, particularly to highly specialized distal enhancer sites^85^.

Here, the finding that a wide range of distinct TFs bind the SWIFT domain of the SMARCD core subunits begins to highlight the biologic diversity encoded by mSWI/SNF complexes and point toward the potential to target or block specialized SWIFT-TF interactions as strategies for therapeutic intervention in cancer and other diseases. Among the >1,600 sequence specific TFs spanning 30+ families and expressed across human tissues that recognize roughly 5–20 bp archetypal DNA motifs^51,86^, specialized TFs are also often grouped by their ability to interact with closed chromatin^46,87,88^. Factors such as PU.1 as well as FOXA1 and GATA3 are considered pioneer factors, a class of TFs capable of accessing 5–20 bp DNA motifs in closed or nucleosome-bound chromatin, coordinating chromatin opening, and allowing the binding of secondary non-pioneer or settler TFs^45,47,82,89^. Pioneer factors are considered the master regulators of lineage-specific gene programs and thus their expression across cell types is tightly regulated and highly cell-type specific^46,88^. Factors belonging to the AP-1 family (FOS, JUN) and NF-kB family (RelA/B) however are expressed more ubiquitously across cell types and are unable to bind nucleosome-bound target sites^44,90,91^. Reports have suggested that pioneer and non-pioneer factors alike may coordinate with mSWI/SNF complexes to achieve cell type specific gene expression^19,44,91^. Our findings here suggest an additional property of pioneer factors such as PU.1 may be to engage directly with SWIFT in an allovalent manner.

We demonstrate that several sequences within the PU.1 activation domain interact with the same receptor site on the SMARCD SWIFT domain. Such allovalent interactions between the transactivation domains of diverse families of TFs and coactivators including mSWI/SNF enable a mechanism for cells to easily evolve TFs with variable affinities for co-activators. In such a model, TFs would evolve higher affinity for mSWI/SNF coactivators through addition of subsequent valency on the activation domains using the existing grammar for the interaction. Consistent with this model, transcription factors with more hydrophobic and acidic residues have higher activation potential. Indeed, several cancers driven by oncogenic fusions of TFs replace their transactivation domains with one containing higher affinity for coactivators including mSWI/SNF. Our results provide a biochemical basis for the observed differences in the transactivation potential of normal and oncogenic transcription factors.

Due to the allovalent interaction between PU.1 and SWIFT, disruption of any single individual peptide-SWIFT interaction would be predicted to be insufficient to completely break TF-remodeler interactions in cells or with full-length proteins incubated in vitro. However, since blocking TF-receptor site on SWIFT domain would impair mSWI/SNF interaction with all polyvalent sites on TFs, SWIFT is an attractive domain for drug discovery efforts aimed to target malignancies driven by TF-driven transcriptional addiction. Indeed, we found that overexpression of the newly characterized SWIFT domain attenuated the proliferation of several TF-addicted cancer cell lines to comparable degrees as treatment with FHD-286, a clinical-grade dual SMARCA4/2 ATPase inhibitor that recently was evaluated in Phase I studies (Fig. 5F, S8G). While antitumoral activity was observed in AML and uveal melanoma settings, development may have been challenged by toxicities associated with systemic full mSWI/SNF family functional inhibition^92,93^. Our findings here open new possibilities for targeted inhibition of TF-addicted cancers via SWIFT domain disruption.

## Materials and Methods

### Cell lines and cell culture

HEK293T lentiX (Clonetech) cells were grown in Dulbecco’s modified Eagle’s medium (Gibco) supplemented with 1% GlutaMax (Gibco), 1% sodium pyruvate (Gibco), 1% mouse embryonic fibroblast non-essential amino acids (Gibco)), 1% Penicillin/Streptomycin (Gibco) with 10% fetal bovine serum (Gibco). Cells were passaged every 3–4 days as described above. Human ASC52telo, hTERT immortalized adipose derived mesenchymal stem cells (MSCs) (ATCC) were grown in mesenchymal stem cell basal medium for adipose, umbilical and bone marrow derived MSCs (ATCC) supplemented with mesenchymal stem cell growth kit for adipose and umbilical-derived MSCs - Low Serum (ATCC) at 37°C, 5% CO_2_, 95% humidity according to the manufacturer’s protocol. Cells were split every 3–4 days and resuspended with fresh medium to maintain culture confluency between 60–80%. To passage, cells were washed with pre-warmed PBS pH 7.4 (Gibco Life Technologies 10010–049) and detached using room temperature TrypLE Express Enzyme (Life Technologies). Cells were passaged with fresh media 20X before a new batch of cells were used. BIN67 cells (gift of Barbara Vanderhyden) were grown in custom media (40% Dulbecco’s modified Eagle’s medium, 40% Ham’s F12 (Gibco), 20% fetal bovine serum (FBS) (Gibco) supplemented with 1% Glutamax (Gibco) and 1% Penicillin-Streptomycin (Gibco)) at 37°C, 5% CO_2_, 95% humidity. MOLM-13 and Ovise cells were grown in RPMI-1640 (Gibco), 10% fetal bovine serum (Gibco), supplemented with 1x Glutamax (Gibo), 1% Penicillin-Streptomycin. ES2 and U2OS cells were cultured in EMEM media (Gibco) with 10% fetal bovine serum (Gibco), supplemented with 1x Glutamax (Gibo), 1% Penicillin-Streptomycin. Cells were maintained by passaging every 4–5 days when cells reached 80–90% confluency by washing with pre-warmed PBS pH 7.4 and detaching cells with Trypsin-EDTA (0.25%) (Gibco).

### Cloning of Mammalian and Bacterial Expression Constructs

All TF expression constructs were PCR-amplified from cDNA or existing expression constructs purchased from Addgene, Dharmacon, or obtained from Harvard University DNA repository and the DNASU Plasmid repository using Phusion High-Fidelity DNA Polymerase (NEB) with GC buffer (NEB) or HF buffer (NEB) and custom PCR primers introducing an in-frame N-terminal HA-tag (YPYDVPDYA). PCR-amplified fragments were gel purified using 1% agarose gel in Tris-Acetate EDTA (TAE) buffer and PCR cleanup columns (Qiagen) and cloned into a modified pTight vector from Clonetech (EFL1-alpha promoter) with puromycin resistance or blasticidin resistance cassette using In-fusion (Clontech) at the Not1 cut site. Recombination products were transformed into One-Shot Stbl3 chemically competent E. coli (Invitrogen) and colonies were selected for plasmid purification (Qiagen) and sequence verified. BRG1 WT-V5 and BRG1 K785R-V5 lentiviral expression constructs in pLEX307 were constructed as previously published(*33*, *68*). EF-1a-MCS-PGK- HA-DPF2 construct used for endogenous cBAF purification was previously described^9,11^. PU.1 deletion mutant constructs were constructed by PCR amplifying fragments flanking the desired deletion with 30 bp of complementarity. N-terminal and C-terminal fragments were amplified separately, gel purified, and fused together using a second PCR with the two fragments as templates. Final fragments were gel purified and cloned into a modified pTight vector (EF1-alpha promoter) containing blasticidin resistance, as above. Single amino acid alanine substitution mutations were generated by site directed mutagenesis (SDM) using Q5 Site-Directed Mutagenesis kit (NEB) according to manufacturer’s recommendations using primers designed by NEBaseChanger (NEB). Constructs for bacterial expression and purification of mutants were cloned into pGOOD with N-terminal GST and C-terminal 6X His tags using In-fusion (Clontech) at the EcoRI cut site. pMD2.G was a gift from Didier Trono (Addgene plasmid # 12259; http://n2t.net/addgene:12259; RRID:Addgene_12259). psPAX2 was a gift from Didier Trono (Addgene plasmid # 12260; http://n2t.net/addgene:12260; RRID:Addgene_12260). Human SWIFT domain sequence was obtained from IDT and cloned into pGOOD with N-terminal 6x-His tag for protein purification from bacterial cells. For protein purification from mammalian cells, SWIFT domain sequence with N-terminal FLAG tag and C-terminal HA-tag was cloned into pCAG plasmid (addgene 11150) using infusion master mix (Clontech).

### Human SMARCD2 SWIFT domain

LTQKRKLRIYISNTFSPSKAEGDSAGTAGTPGGTPAGDKVASWELRVEGKLLDDPSKQKRKFSSFFKSLVIELDKELYGPDNHLVEWHRMPTTQETDGFQVKRPGDLNVKCTLLLMLDHQPPQYQHPDPIVINHVISVDPNDQKKTACYDIDVEVD

### Lentiviral Production and Transduction

Lentiviral particles were produced by transfecting HEK293T LentiX (Clontech) cells plated to 80% confluency using 1 μg mL^−1^ PEI (Polysciences, Inc) and lentiviral expression vector and packing vectors psPAX2 and pMD2.g in ratio of 4:3:1 respectively as previously described^95,96^. 72 hours after transfection, media was filtered through 0.45 μm filter (EMD Millipore SE1M003M00) and viral particles were concentrated by ultracentrifugation at 20,000 rpm for 2.5 hrs at 4°C using a SW32Ti rotor. Viral particles were resuspended in PBS and used to transduce cells plated to 70% confluency using 8 μg ml^−1^ polybrene (Santa Cruz Biotechnology) or 10 μg mL^−1^ protamine sulfate (Sigma-Aldrich, P4020–5G) for BIN-67. Media was exchanged 24 hours following virus addition and cells were selected with 8 μg mL^−1^ blasticidin or 1 μg mL^−1^ puromycin starting 48 hours after virus addition. Expression of desired constructs was verified by western blot and cells were harvested for genomics based analyses on Day 7 post-transduction.

### Nuclear Extraction and Immunoprecipitation

Nuclear protein extracts were prepared using standard techniques. Briefly, cells were washed with cold PBS to remove media. Pelleted cells were resuspended in hypotonic buffer (EBO: 50mM Tris pH7.5, 0.1% N-40, 1 mM EDTA, 1 mM MgCl2, supplemented with protease inhibitors (10 μg mL^−1^ each chymostatin, pepstatin, leupeptin (EMD Millipore) in DMSO and 1mM PMSF (Gold Bio) in isopropanol). Nuclei were pelleted at 5,000 rpm for 5 min at 4°C. Nuclei were resuspended in high salt buffer (EB300: 50mM Tris pH 7.5, 300mM NaCl, 1% NP-40, supplemented with protease inhibitors as above). Lysates were incubated on ice for 10 min and sonicated using 3–6 pulses of 10 seconds with a Misonix Sonicator 3000 Ultrasonic Cell Disruptor system with microtip probe with max power output of 6W. Lysates were pelleted for 5 min at max speed in a benchtop centrifuge and supernatant was quantified using BCA assay reagents (Pierce). 0.5–1 mg of protein per condition were supplemented with 1 mM DTT (Sigma-Aldrich) and was used for immunoprecipitation with 3–5 ug of antibodies overnight at 4°C with rotation. 30 μl of Protein-G Dynabeads were added and incubated for 2 hours with rotation and washed with EB150 (EB300 with 150 mM NaCl) 6 times and eluted with 2X LDS loading dye with 1 mM DTT at 95°C for 5 min and loaded on SDS-PAGE gel. Whole cell lysates were prepared using 5X pellet volume of protein extraction buffer (20mM Tris, 1.5% SDS). Lysed cells were incubated at 95°C for 2 min to denature DNA, sonicated and quantified as above.

### SDS-PAGE and western blotting

Western blotting was performed using standard techniques. Briefly samples were loaded on 4–12% Bis-Tris Plus SDS-PAGE gels (Invitrogen) and run at 80V for 20 min, followed by 130V for 1 hour. Proteins were transferred to polyvinylidene difluoride (PVDF) membranes (Immobilon-FL, EMD Millipore) for 90 min at 30V and blocked for 1 hr at room temperature in 5% milk (Boston BioProducts) in PBS. Blots were incubated O/N with primary antibodies, washed 3X in PBST and incubated with secondary fluorophore-conjugated species-specific antibodies (Li-Cor) for one hour. Membranes were washed 3X with PBS-T and 1X with PBS and visualized using Li-Cor Odyssey CLx.

### ChIP-seq sample preparation and sequencing

ChIP experiments were performed per standard protocols (Millipore) with minor modifications. Briefly, cells were cross-linked for 10 min with 1% formaldehyde at 37°C and quenched with 125 mM glycine. Ten million fixed cells were used per chromatin immunoprecipitation experiment. This reaction was subsequently quenched with 125 mM glycine for 5 min. Chromatin from isolated nuclei was fragmented using Covaris E220 ultrasonicator (Covaris) to 200–700 bp size. Primary antibodies were incubated O/N with sonicated chromatin, washed, and eluted. Eluted material was treated with RNAse A (ThermoFisher Scientific) and Proteinase K (ThermoFisher Scientific), and purified using AMP Pure beads (Beckman Coulter). DNA was quantified using Qubit dsDNA HS Assay Kit (ThermoFisher Scientific). Libraries were prepared for next-generation sequencing using NEBNext Ultra II DNA library Pep kit (NEB). Libraries were quantified using Qubit dsDNA HS Assay Kit (ThermoFisher Scientific) and Kapa library quantification kit for Illumina platforms (Kapa Biosystems) and appropriate size distribution was verified using TapeStation High Sensitivity D1000 screentape and reagents (Agilent Technologies). Samples were pooled, denatured according to standard protocols from Illumina and sequenced with single-end reads using 75 cycle Nextseq 500/550 High Output Kit v2 for Illumina NextSeq 500 (Illumina).

### CUT&RUN

CUT&RUN was performed as previously described with modifications(*87*, *88*). MSCs were harvested 7-days post transduction with HA-TF lentivirus. Cells were dissociated using TrypLE Express (ThermoFisher) and washed once with PBS pH 7.4. Cells were counted with Countess II (Invitrogen). 500,000 live cells were used per epitope as determined by Trypan Blue exclusion. Cells were washed 1X with Wash Buffer (20 mM HEPES pH 7.6, 150 mM NaCl, 0.5 mM Spermidine and supplemented with protease inhibitors (10 μg mL^−1^ each chymostatin, pepstatin, leupeptin in DMSO)). Washed cells were incubated with 10 μL each activated magnetic Concanavalin A beads (Polysciences) (activated by washing 2X with Binding Buffer (20mM HEPES pH 8.0, 10 mM KCl, 1 mM CaCl_2_, 1mM MnCl_2_ at RT) for 10 min at room temperature with rotation. Cells bound to beads were resuspended in Antibody Buffer (Wash Buffer, 0.1% Triton X-100, 2 mM EDTA) and incubated with desired antibody for 2 hours at 4°C (**Table S2.10**). Beads were washed 1X with Triton X-100 Buffer (Wash Buffer, 0.1% Triton X-100), resuspended in Triton X-100 Buffer with 3 ng/μL purified pA-MNase and incubated with rotation for 1 hour at 4°C. Beads were washed 2X with Triton X-100 buffer to remove unbound pA-MNase and resuspended in Triton X-100 buffer. pA-MNase was activated by adding 2mM CaCl_2_ and beads were incubated in an ice water bath for 30 min. Equal volume of 2X Stop buffer (340 mM NaCl, 20 mM EDTA, 4 mM EGTA, 0.2% Triton-X 100, 100 μg/mL RNase A (Thermo Fisher Scientific), 50 μg/ml Glycogen (Thermo Fisher Scientific), 2 pg/mL yeast spike-in DNA (Cell Signaling)) was added to halt MNase activity. Cut fragments were released from insoluble nuclear chromatin by incubation at 37°C for 20 min. Released DNA fragments were treated with Proteinase K (ThermoFisher Scientific) for 1 hour at 55°C and purified by mixing samples with equal volumes phenol chloroform isoamyl alcohol (Invitrogen), vortexing and applying sample to MaXtract High Density phase lock tubes (Qiagen). Samples were further purified by mixing with equal volumes chloroform (Sigma) and purified aqueous phase containing DNA was concentrated by ethanol precipitation with 2 μL of 5 mg/mL glycogen (ThermoFisher Scientific) and quantified using Qubit dsDNA HS Assay Kit (ThermoFisher Scientific).

### ATAC-seq Sample Preparation

ATAC-seq was performed as previously described^97^ with 50,000 cells per sample. Cells were dissociated and counted, as described above, 7-days post transduction with lentivirus. Cells were washed with cold PBS, resuspend in lysis buffer (10mM Tris-HCl pH7.4, 10 mM NaCl, 3 mM MgCl_2_, 0.1% IGEPAL) and centrifuged at 500 g for 10 min at 4°C. Nuclei were resuspended in transposition mix (1X TD Buffer, 2.5 ul TDE1 Nextera Tn5 Transposase (Illumina)) and incubated with mild rotation for 30 min at 37°C. Tagmented DNA was purified using MinElute PCR cleanup kit (Qiagen). Samples were amplified using 10 total PCR amplification cycles with custom PCR Primers using NEBNext HF PCR Master Mix (NEB). Samples were purified with 1.2X Agencourt AMPure XP beads (Beckman Coulter) and size distribution was determined using D5000 HS reagents and screentapes (Agilent) on 2200 TapeStation Instrument (Agilent). Samples were pooled in equimolar amounts and sequenced on Illumina Next-seq 500 with NextSeq 500/550 High Output Kit V2 75 cycles (Illumina) using 37bp paired-end sequencing parameters.

### RNA-seq Sample Preparation

Cells were dissociated and counted, as described above, 7-days post transduction with lentivirus. 2 × 10^6^ live cells as were washed with PBS and RNA was extracted using QIAshredder and RNeasy Mini Kit (Qiagen) per the manufacturer’s instructions. 1 μg of total RNA was used as input for library preparation and processed per the manufacturer’s instructions using NEBNext Poly(A) mRNA magnetic isolation module (NEB) followed by the NEBNext Ultra II Directional RNA library prep kit for Illumina (NEB) with NEBNext Multiplex Oligos for Illumina (NEB). Samples were collected in biological replicate. ERCC synthetic RNA spike-in control (Thermo Fisher Scientific) was added at the beginning of library preparation per manufacturer’s recommendations to normalize samples across conditions. Libraries were quantified using Qubit dsDNA HS Assay Kit (Thermo Fisher Scientific) and size distribution was verified using Tapestation D1000 HS reagents and screentapes (Agilent). Equimolar amounts of libraries were pooled, denatured, and sequenced on Illumina Next-seq 500 with NextSeq 500/550 High Output Kit V2 75 cycles (Illumina) according to standard Illumina protocols with single-end sequencing parameters.

### CUT&TAG

CUT&TAG was performed following published protocols (Epicypher)^98^. Cells were dissociated from culture plates using TrypLE Express (Thermo Fisher Scientific) and washed once with PBS pH 7.4 (Gibco). Cells were counted with Countess II (Invitrogen). 200,000–500,000 live cells were used per epitope as determined by Trypan Blue (Thermo Fisher Scientific) exclusion. Cells were processed for Cut & Tag as previously published^99^ and according to manufacturer’s CUTANA protocol (Epicypher) with the use of BioMag Plus Concanavalin A beads (Polysciences), CUTANA pAG-Tn5 (Epicypher), primary antibodies, and Guinea Pig anti-Rabbit IgG (Heavy & Light chain) antibody (Antibodies-Online). Tn5 transposed fragments were amplified between 14–18 cycles using NEB Next HF 2X PCR Master Mix (NEB) and custom primers originally designed for ATAC-seq library amplification to allow for multiplexing of 50+ samples. Amplified DNA was purified using 0.75X AMPpure Beads (Beckman Coulter) to remove primer-dimers. Libraries were quantified and QC’d as above. Samples were sequenced by the Dana Farber Cancer Institute Molecular Biology Core Facility using paired-end read parameters on an Illumina Nextseq 500 (Illumina) using Illumina NextSeq 500/550 High Output Kit V2 75 cycles (Illumina).

### Protein Purification from Mammalian Cells

Purification of endogenous mammalian cBAF complexes using HA-DPF2 as bait and purified HA-PU.1 was performed as previously described^11^ using hypotonic followed by high salt lysis to extract nuclear proteins. Briefly, stable cell lines expressing lentivirally transduced HA-tagged baits were cultured in 150mm dishes and expanded to 300 (HA-DPF2) or 150 (HA-PU.1) total plates. Cells were scraped and washed with cold PBS. Cells were lysed in hypotonic buffer (HB: 10 mM Tris HCl pH 7.5, 10 mM KCl, 1.5 mM MgCl_2_ 1 mM DTT, and 1 mM PMSF) for 5 min on ice and centrifuged at 5000 rpm for 5 min at 4°C. Pellets were resuspended in HB buffer supplemented with protease inhibitor cocktail and cells were lysed using glass Dounce homogenizer. Lysed cells were centrifuged at 5000 rpm for 20 min at 4°C and nuclei were resuspend in High Salt Buffer (HSB: 50mM Tris HCl pH 7.5, 300 mM KCl, 1mM MgCl_2,_ 1 mM EDTA, 1% NP40, 1mM DTT, 1 mM PMSF and protease inhibitor cocktail) and incubated with rotation for 1 hour at 4°C followed by centrifugation to remove chromatin pellet at 20,000 rpm for 1 hour at 4°C using SW32Ti rotor. Chromatin pellet was removed and supernatant was filtered through Advantec Grade QR200 Quartz Fiber Filters (Cole-Parmer). Complexes were purified from clarified nuclear extracts using magnetic HA beads (Pierce) overnight at 4°C. HA beads were washed 6X with HSB and purified proteins were eluted using Elution Buffer (HSB + 1 mg/ml HA peptide (GenScript) 4X for 1.5 hours each at 4°C. Eluted proteins were subjected to silver staining and density gradient centrifugation for analysis of yield and purity.

### Protein Expression and purification of TFs

Transcription factors (PU.1, FOS, and POU2F3) for in vitro binding experiments was purified from 293-ExpiF cells (thermo scientific; Cat# A14527), grown in Expi media (thermo scientific; Cat# A1435102). 800 μg of plasmids (pCAG containing gene of interest) were transfected into 2 billion cells in 1 L culture using 4 mg of polyethyleminine. After 16 hours, valproic acid, sodium propionate, 1x NEAA were added to culture. Cells were harvested after 2 days by centrifugation at 1000 g for 10 min, washed with 50 ml of PBS and resuspended in lysis buffer (20 mM HEPES pH 8.0, 1 mM EDTA, 300 mM KCl, 1% NP-40, 1x protease inhibitor cocktail, 0.5 mM DTT, 0.4 mM PMSF). After protein extraction for 1 hour and clarification using centrifugation at 20,000 g for 30 min, whole cell extract was incubated with 0.7 ml of FLAG M2 magnetic beads (Sigma; Cat# M8823) for 2 hours. Beads bound to proteins of interest were washed 6-times with binding buffer and proteins were eluted using 3x FLAG peptides.

Eluted proteins were further purified by anion exchange chromatography using a custom Capto-Q column (0.15 ml column volume) and eluted by a KCl (0–1 M) over 25 column volumes. Fractions containing proteins of interested were pooled and further purified by size-exclusion chromatography using superdex 200 (Cytiva; Cat# 28-9909-44). Fractions containing protein of interest were pooled and used for downstream experiments.

### Peptide Pulldowns

Peptide pulldowns were performed as previously described^100^. 3 nmol of biotinylated PU.1 peptides (novopep, custom) were incubated with 0.3 nmol of purified HA-tagged SMARCD2 SWIFT or AF9 YEATS domains overnight in binding buffer containing 20 mM HEPES, 1 mM EDTA, 75 mM KCl, 0.01% tween-20. 25 μl of streptavidin conjugated dynabeads (cat# 65601) for 2 hours to capture biotinylated peptides. Dynabeads were washed 7-times with binding buffer and boiled in 2x Laemmli buffer to elute captured proteins. After removal of dynabeads, eluates were analyzed by immunoblotting using HA antibodies (Cell Signaling; Cat# 3724).

### Nuclear extract preparation and SWIFT-pulldown experiments

For SWIFT pulldown experiments, 1–2 billion cells were resuspended in hypotonic lysis buffer (20 mM HEPES pH 8.0, 1 mM EDTA, 4 mM MgCl2, 10 mM KCl, 1x protease inhibitor cocktail, 1 mM DTT, 0.4 mM PMSF). Nuclei were resuspended in 10 ml of buffer-AB (20 mM HEPES pH 8.0, 110 mM KCl, 1 mM EDTA, 2 mM MgCl2, 1x protease inhibitor cocktail, 0.4 mM PMSF, 1 mM DTT). 1.1 ml of 4 M ammonium sulfate was added to nuclei suspension. After 1 hour incubation, chromatin fraction was pelleted by ultracentrifugation in Ti-45 rotor at 35k RPM for 90 min. Nuclear proteins were salted out by slowly adding 0.3 g of powdered ammonium sulfate to the clarified extract followed by ultracentrifugation at 20K RPM in Ti-45 rotor for 30 min. Pellet containing nuclear proteins were resuspended in binding buffer containing 20 mM HEPES pH 8.0, 150 mM KCl, 1 mM EDTA, 0.5 mM DTT, 1x protease inhibitor cocktails, 0.4 mM PMSF. Purified N-terminally HA-tagged SWIFT domain was incubated with nuclear extract at a final concentration of 200 nM for 2 hours at 4 °C. Following binding, HA-antibody conjugated dynabeads (Thermo scientific; Cat# 88837), pre-blocked with BSA were added to the mixture for 2 hours at 4 °C. Beads were washed 6 times in binding buffer and stored in 200 mM HEPES pH 8.0 until mass-spectrometry.

### Density sedimentation gradients

Eluted protein complexes were separated by density using 10%–30% glycerol gradients prepared using Gradient Master containing 25mM HEPES pH 7.9, 0.1mM EDTA, 12.5mM MgCl2, 100mM KCl supplemented with1mM DTT and protease inhibitors as previously described^9,11^. Briefly, eluted proteins were loaded on top of 11 ml gradient and centrifuged at 40,000 rpm for 16 hours at 4°C. Individual fractions (21 × 550 μl) were manually aspirated from the top of the gradient. 80 μL of each collected fraction were concentrated using 10 μL of Strataclean beads (Agilent), eluted with 2X NuPage LDS sample buffer (ThermoFisher Scientific), loaded onto SDS-PAGE gels and stained using Silver Quest Silver Staining Kit (ThermoFisher Scientific) according to manufacturer’s protocol. Fractions with confirmed purified expression of desired protein/complex were pooled and concentrated using appropriate molecular weight cutoff protein concentrator (Pierce). Samples were snap frozen and kept at −80°C until needed.

### Electrophoretic Mobility Shift Assay

The protein-DNA binding reaction was carried out as previously published with a few modifications^101^. Short DNA probes containing PU.1 DNA binding motif or scrambled control were ordered as custom single stranded oligos from Integrated DNA Technologies (IDT) and annealing was carried out on a thermocycler with the appropriate DNA complement. Purified HA-PU.1 was titrated and incubated with constant X amount of annealed oligo. The DNA binding assay was performed in a total volume of 10 μL of binding buffer (contents) at room temperature for 30 min after which reaction was mixed with 10X DNA loading dye and run on 8% Novex TBE gel (ThermoFisher Scientific) in 1X TBE buffer at 100V for 1 hour followed by 30 min staining with 1X SybrGold in TBE and visualization using DNA station.

### GEE labeling

Differential GEE footprinting was performed on purified cBAF complexes, cBAF complexes incubated with nucleosome core particle (NCP), and cBAF complexes incubated with purified PU.1. The molar ratios for cBAF complexes with nucleosome and PU.1 were 1: 5 and 1: 8, respectively. To reach the binding equilibrium, cBAF complexes and its binding partners were incubated under 4°C overnight, prior to the footprinting. For GEE labeling, glycine ethyl ester (GEE), 1-ethyl-3-(3-dimethylaminopropyl) carbodiimide (EDC) stock solutions were prepared fresh in PBS buffer. GEE was added to each pre-equilibrated samples, followed by addition of EDC which initiates the footprinting reaction. The final concentrations of protein, GEE, and EDC were 250 nM, 20 mM, 1 mM, respectively. The reaction was carried out at 25°C for 1 h, before quenching by addition of equal volume of 1 M ammonium acetate. The proteins were then immediately purified by acetone precipitation. Labeling experiments were performed in triplicate n=3.

### Proteolysis of GEE labeled peptides

Urea (8M) was added to dissolve the acetone-precipitated protein pellets. The proteins’ cysteine residues were then reduced and alkylated with TCEP and iodoacetamide (IAM), respectively. Lys-C and trypsin were next successively added with a enzyme-to-protein ratio of 1:20 and 1:5 (w:w), respectively. Samples were incubated at 37 °C with Lys-C for 6 h and trypsin for 12 h. The digestion was quenched by adding formic acid to a final concentration of 5% (by volume).

### Mass spectrometry analysis of GEE labeled peptides

A Dionex UltiMate 1000 system (Thermo Fisher Scientific) was coupled to an Orbitrap Fusion Lumos (Thermo Fisher Scientific) through an EASY-Spray ion source (Thermo Fisher Scientific). Nanoflow liquid chromatography separation was carried as previously described(*95*). Briefly, peptide samples were loaded (15 μl min^−1^; 1 min) onto a trap column (100 μm × 2 cm; 5 μm Acclaim PepMap 100 C18; 50 °C), eluted (0.2 μl min^−1^) onto an EASY-Spray PepMap RSLC C18 column (2 μm; 50 cm × 75 μm ID; 50 °C; Thermo Fisher Scientific) and separated with the following gradient (all % buffer B (that is, 0.1% formic acid in acetonitrile)): 0–110 min: 2–22%; 110–120 min: 22–35%; 120–130 min: 35–95%; 130–150 min: isocratic at 95%; 151–153 min: 95–2%; 153–171 min: isocratic at 2%. The spray voltage was 1,900 V, the ion transfer tube temperature was 275 °C and the RF lens was 30%. Mass spectrometry scans were acquired in profile mode and tandem mass spectrometry scans were acquired in centroid mode, for ions with charge states 2–7, with a cycle time of 3 s. Mass spectrometry spectra were recorded from 375–1,500 Da at 120-K resolution (at *m/z* 200), and higher-energy collisional dissociation tandem mass spectrometry was triggered above a threshold of 2.0 × 10^4^, with quadrupole isolation (0.7 Da) at 30-K resolution and a collision energy of 30%. Dynamic exclusion was used (60 s), and monoisotopic precursor selection was on.

### GEE labeling mass -spectrometry data processing

Identification of the unmodified peptides and assigned modifications were done by using Byologic (Protein Metrics) and further validated by manual inspection. Modification sites were identified based on MS/MS. Signal intensities of the unmodified peptide (Iu) and its modified species (Iox) were integrated using Byologic (Protein Metrics) from the extracted ion chromatograms (XICs). The GEE modification fraction of a residue was calculated using the following equation: % modified = I_modified_/(I_modified_ + I_unmodified_)×100. Quantification of the modified species was based on the GEE products and its hydrolyzed products (+85.0522 Da, +57.0209 Da

### Mapping changes in GEE exposure onto 3D mSWI/SNF structures

Changes in exposure between the bound state (cBAF+NCP or BAF+PU.1) and unbound state (cBAF only) were visualized on published cryoEM structure cBAF complex. Change values were extended in windows of 11aa centered on each GEE label and values were averaged in overlapping windows. Ambiguous GEE labels (labels with more than one candidate D/E site) were split into multiple observations and preprocessed similarly. GEE labels from paralogs not represented on the structure (ACTA2, ARID1B, BCL7B, BCL7C, SMARCD2, SMARCD3) were remapped to the primary paralog in the structure (ACTA2:ACTB, ARID1B:ARID1A, BCL7B:BCL7A:, BCL7C:BCL7A, SMARCD2:SMARCD1, SMARCD3: SMARCD1), and preprocessed and visualized similarly. Changes in exposure were stored in the b-factor column of the PDB file and visualized using the third-party color_b PyMOL script as a blue-white-red heatmap clipped at −30% (dark blue) to 30% (bright red).

### Mapping changes in GEE exposure to cBAF complex subunit protein schematics

Changes in exposure between the bound state (cBAF+NCP or cBAF+PU.1) and unbound state (cBAF only) were preprocessed as described in 3D Structure. However, paralogs were not remapped to the primary paralog resolved in the structure of the endogenous cBAF complex to show paralog-specific effects (ARID1A vs ARID1B, SMARCD1 vs SMARCD2, SMARCC1 vs SMARCC2). Change values were visualized as vertical lines across the length of each BAF subunit (blue as a loss in exposure and red as a gain in exposure upon substrate binding). Changes in exposure in cBAF-NCP and cBAF-PU.1 data for the cBAF-NCP structure were merged into the same plot using matplotlib.

### cBAF-NCP and cBAF-^79,101^GEE Overlap Calculations

Changes in exposure visualized across the lengths of the cBAF subunits were analyzed by eye to define merged sites (windows that were within 10–20aa of each other with consistent effects were merged) of exposure. Merged windows of exposure change less than 1% were omitted as noise. Overlapping merged sites of exposure with consistent effects (i.e. both gains in exposure or loss of exposure) when bound to either substrate, NCP and PU.1, were considered overlapped sites of exposure. The remaining sites were binned into disjoint sets of cBAF+NCP exclusive sites and cBAF+PU.1 exclusive sites. These counts were visualized as a Venn diagram using matplotlib.

### Distribution of Exposure between the ATPase and Core Modules

Changes in exposure were filtered to the most significant sites using a 5% cutoff for cBAF+NCP and cBAF+PU.1. Gains and losses in exposure within the Core and ATPase modules for both GEE datasets were tallied and reported as proportions using stacked bar charts in matplotlib.

### RNA-seq Data Analysis

RNA-Seq reads were demultiplexed using bcl2fastq v2.20.0.422 aligned to the hg19 genome with STAR v2.5.2b^102^. Upregulated and downregulated genes were determined using DESeq2 (log2FC = 1, B-H p-value = 0.05)^103^. DESeq2’s estimateSizeFactors function was used to generate normalized counts^104^. ggplot2 was used to generate volcano plots for changes in expression visualized as scatter plots. The eulerr R package was used to generate venn diagrams of differential genes. ComplexHeatmap R package was used to generate heatmaps and visualize Z-Score normalized read counts for each gene across all conditions for each cell line. The Kendall and Spearman distance functions were used to perform hierarchical clustering in heatmaps. clusterProfiler GSEA function^105^ was used through the msigdbr R package to perform GSEA analysis using the Hallmark and C2 gene sets. The “stat” output from DESeq2 was used to determine Differential expression ranking. MOLM13 sgPU.1 RNA data was downloaded from GEO series GSE186131^94^.

### ATAC-seq Data Analysis

ATAC-seq reads were first trimmed using fastp v0.24.0, aligned with Bowtie2 v2.2.9 to the GRCh38 reference genome, and filtered using SAMtools v0.1.19 to remove duplicates and for quality (-F 256 -f 2 -q 30)^106–108^. Reads mapping to regions defined in the ENCODE project’s unified GRCh38 blacklist bed file were removed using bedtools v2.30.0[14]. ATAC-seq data was processed by merging technical replicates using SAMtools merge. Peaks were called using MACS3 (-f BAMPE -g hs -q 0.001 --nomodel --extsize 200 –broad)^109^.

Differential accessibility was determined by calculating reads-per-million values across a merged set of peaks for a given comparison set, and using an RPM fold-change cutoff of 1.5. Differential accessibility was then visualized with venn diagrams generated using eulerr. Site centers with flanking windows of 200bp (total window size of 400bp) were identified across given FASTA sequence sites. Motif enrichment across these sets of sites was determined using HOMER findMotifsGenome.pl against genone-background (-size 400) unless otherwise noted. Barplots were generated to visualize HOMER motif results using ggplot2. Heatmaps and deepTools computeMatrix as used to generate metaplots over indicated peaks^110^. deepTools bamCoverage (--binSize “40” --normalizeUsing “CPM” --exactScaling) was used to generate bigwig inputs for heatmaps. Stacked bar charts generated with ggplot in R to highlight distance to TSS highlighting the proportion of promotor, promoter proximal and distal enhancer regions, determined using bedtools and the GENCODE v46 annotations^111^.

### CUT&RUN-seq and CUT&TAG-seq Data Analysis

ChIP-seq reads were aligned in a manner identical to ATAC-seq reads. Peaks for CUT&RUN were generated with MACS2 (-f BAM -g hs -q 0.01 --nomodel). Peaks for CUT&TAG were generated using SEACR (at a q value of 0.01, using the “non” normalized and “stringent” settings)^112^.

FASTA sequences across these sets of sites were generated using site centers with flanking windows of 200bp (total window size of 400bp). Enriched motifs across these sets of sites were determined using HOMER findMotifsGenome.pl against genome-background (-size 400) unless otherwise noted. Bigwigs were generated using deepTools and rendered using karyoplotR.

### AP-MS Analysis

Differential proteins from AP-MS data were identified using DeqMS and plotted via ComplexHeatmap and ggplot2. Differential transcription factors were identified using the list of transcription factors published in Lambert et al^51^.

### GTEX and CCLE Analysis

Analysis of CCLE and DepMap CRISPR screening data was conducted using the public 25q2 release^35,113^. Analysis of GTEX expression data was conducted using version 10.

### Cryo-EM Structures and Alphafold Predicted Structures

Resolved Cryo-EM structures for 9A0K, 6LTJ, 4TMP, 3DO7, and 1FRG were downloaded from PDB and rendered using Pymol^9,57–59,66^. Electrostatic surfaces were also generated using Pymol. Alphafold2 predictions for the proteome were used to determine structural domains, and were also used to add unresolved domains to 6LTJ via alignment^58^.

Alphafold3 predictions were used to predict affinity between the SMARCD2 SWIFT domain and PU.1 sequences^114–117^. Predictions were parsed to extract the contact probability between each residue within a subsequence and L256, I258, V272, W274, F286, and V288 of SMARCD2, and aggregated as a log2 mean probability, which was then averaged across all sequence predictions containing that residue. Alphafold3 was accessed at alphafoldserver.com

### Sequence and Structure Alignment

Sequence alignment was conducted using Clustalw^118^ using the primary isoform of the SwissProt entry for a given protein. Sequence conservation was determined using the sequence of the isolated SMARCD2 SWIFT domain and Consurf. Structural similarity was determined using Foldseek^119^ to search for the Alphafold3 predicted structure of the SMARCD2 SWIFT domain against the Uniprot/Proteome database. Protein hits identified in this manner were then aligned against each other in an all-to-all fashion to obtain a TMscore, which was then used to perform hierarchical clustering and displayed using dendextend^120^.

## Supplementary Material

Supplement 1
