# Supplementary material for: A SWI/SNF-specific Ig-like domain, SWIFT, is a transcription factor binding platform
Source: bioRxiv. 2025 Aug 1:2025.08.01.667725. Preprint. [Version 1] doi: 10.1101/2025.08.01.667725 (PMC12324477; doi:10.1101/2025.08.01.667725)
Supplement: Supplement 1 [file media-1.pdf]

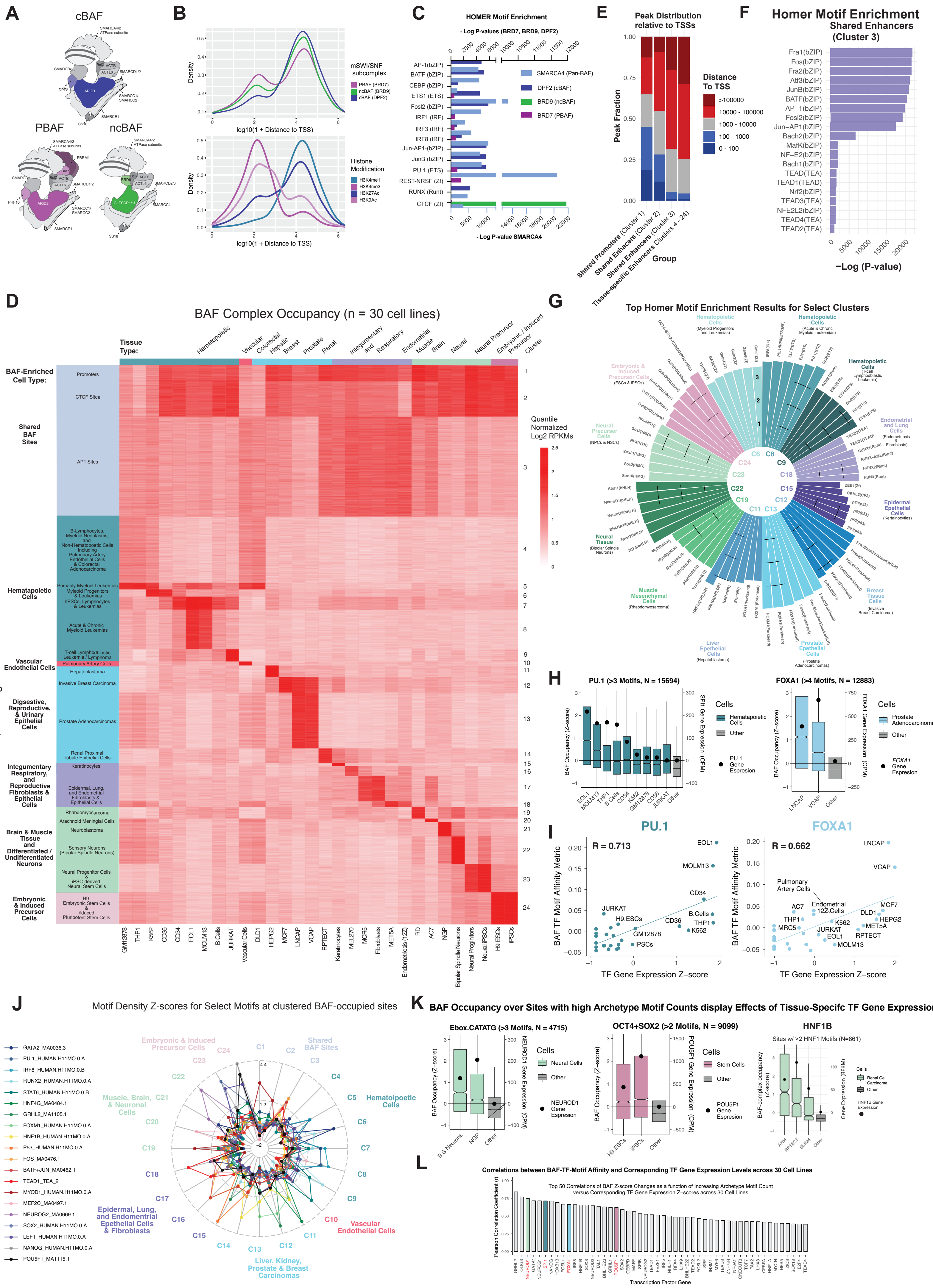
A
cBAF
PBAF
ncBAF
B
mSWI/SNF subcomplex
PBAF (BRD7)
ncBAF (BRD9)
cBAF (DPF2)
Histone Modification
H3K4me1
H3K4me3
H3K27Ac
H3K9Ac
log10(1 + Distance to TSS)
Density
C
HOMER Motif Enrichment
- Log P-values (BRD7, BRD9, DPF2)
SMARCA4 (Pan-BAF)
DPF2 (cBAF)
BRD9 (ncBAF)
BRD7 (PBAF)
- Log P-value SMARCA4
E
Peak Distribution relative to TSSs
Distance To TSS
>100000
10000 - 100000
1000 - 10000
100 - 1000
0 - 100
Peak Fraction
Shared Promoters (Cluster 1)
Shared Enhancers (Cluster 2)
Shared Enhancers (Cluster 3)
Tissue-specific Enhancers Clusters 4 - 24)
Group
F
Homer Motif Enrichment
Shared Enhancers (Cluster 3)
−Log (P-value)
D
BAF Complex Occupancy (n = 30 cell lines)
Tissue Type:
BAF-Enriched Cell Type:
560,948 Merged mSWI/SNF Peaks
Quantile Normalized Log2 RPKMs
Cluster
G
Top Homer Motif Enrichment Results for Select Clusters
H
PU.1 (>3 Motifs, N = 15694)
FOXA1 (>4 Motifs, N = 12883)
BAF Occupancy (Z-score)
SPI1 Gene Expression (CPM)
FOXA1 Gene Expression (CPM)
Cells
Hematopoietic Cells
Prostate Adenocarcinoma
Other
I
PU.1
FOXA1
R = 0.713
R = 0.662
BAF TF Motif Affinity Metric
TF Gene Expression Z−score
J
Motif Density Z-scores for Select Motifs at clustered BAF-occupied sites
K
BAF Occupancy over Sites with high Archetype Motif Counts display Effects of Tissue-Specifc TF Gene Expression
Ebox.CATATG (>3 Motifs, N = 4715)
OCT4+SOX2 (>2 Motifs, N = 9099)
HNF1B
L
Correlations between BAF-TF-Motif Affinity and Corresponding TF Gene Expression Levels across 30 Cell Lines
Top 50 Correlations of BAF Z-score Changes as a function of Increasing Archetype Motif Count versus Corresponding TF Gene Expression Z-scores across 30 Cell Lines
Pearson Correlation Coefficient (r)
Transcription Factor Gene

Figure S1. **Unique sequence-specific transcription factor motifs underlie cell type-specific mSWI/SNF complex occupancy at distal enhancers genome-wide.** A. Schematic depicting the subunit compositions of cBAF, PBAF and ncBAF complexes. B. Density plots showing the distribution of cBAF, PBAF and ncBAF peaks (top) and histone modifications (bottom panel; H3K4me3, H3K4me1, H3K9ac and H3K27ac) as a function of distance from annotated transcription start sites (x-axis). **C**. Homer motif enrichment -Log (p-value) for top 15 enriched transcription factor motifs over sites occupied genome-wide by SMARCA4 (pan-BAF), DPF2 (cBAF), BRD7 (PBAF) and BRD9 (ncBAF) by ChIP-seq in EOL1 cell line. **D**. Unsupervised clustering of 560,948 merged BAF complex occupied ChIP-seq peaks (SMARCA4/SMARCC1) genome-wide across 30 cell lines. Cell lines are annotated by tissue type and/or cancer subtype. **E**. Distance from TSS of mSWI/SNF peaks in clusters defined in D. **F.** Homer motif enrichment -Log (p value) for top 15 enriched transcription factor motifs over sites in cluster 3 as defined in D. F. HOMER motif analysis of shared enhancers in cluster 3 from D. **G.** Homer motif enrichment (log (p-value)) of top transcription factor motifs in tissue-specific mSWI/SNF clusters defined in D. **H**. BAF complex occupancy (Z-score) at sites with >3 TF motifs for PU.1, and >4 TF motifs for FOXA1 in cell lines of shared lineaage where corresponding gene expression of TF >1 CPM. . Expression of the PU.1 and FOXA1 in the cell lines is marked in black circles. **I.** Correlation between BAF occupancy as a function of corresponding TF motif archetype counts (Zscore(SMARCA4 ChIP-seq RPKM / [Motif])) for motifs of PU.1 and FOXA1, respectively in cell lines of shared lineages and their expression (z-score normalized). **J.** Density of select motifs of transcription factors (z-score normalized) at various clusters of BAF complex occupancy sites. **K.** BAF complex occupancy (Z-score) at sites with >3, >2, and >2 TF motifs for NEUROD1, OCT4/SOX2 and HNF1B, respectively in cell lines of shared lineaage where corresponding gene expression of TF >1 CPM. **L**. Pearson correlation coefficient (r) between TF gene expression (Zscore RPKM) and BAF occupancy as a function of corresponding TF motif archetype counts (Zscore(SMARCA4 ChIP-seq RPKM / [Motif])) across unique enhancer sites from B (C4-24) with r>0.5.

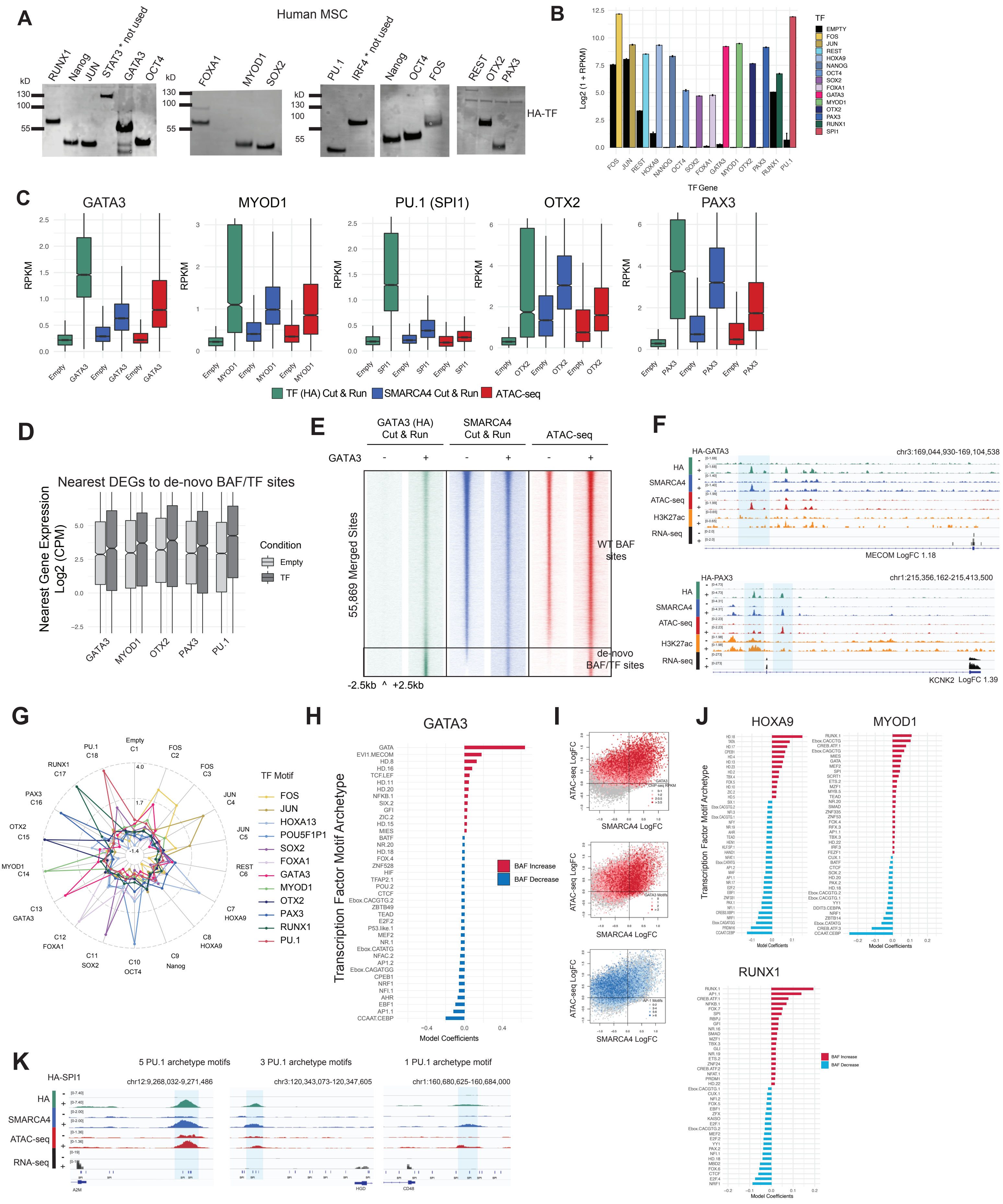
A
Human MSC
RUNX1
Nanog
JUN
STAT3 * not used
GATA3
OCT4
FOXA1
MYOD1
SOX2
PU.1
IRF4 * not used
Nanog
OCT4
FOS
REST
OTX2
PAX3
kD
130
100
55
HA-TF
B
Log2 (1 + RPKM)
TF Gene
TF
EMPTY
FOS
JUN
REST
HOXA9
NANOG
OCT4
SOX2
FOXA1
GATA3
MYOD1
OTX2
PAX3
RUNX1
SPI1
C
GATA3
MYOD1
PU.1 (SPI1)
OTX2
PAX3
RPKM
TF (HA) Cut & Run
SMARCA4 Cut & Run
ATAC-seq
D
Nearest DEGs to de-novo BAF/TF sites
Nearest Gene Expression Log2 (CPM)
Condition
Empty
TF
E
GATA3 (HA) Cut & Run
SMARCA4 Cut & Run
ATAC-seq
GATA3
55,869 Merged Sites
WT BAF sites
de-novo BAF/TF sites
-2.5kb ^ +2.5kb
F
HA-GATA3
chr3:169,044,930-169,104,538
HA
SMARCA4
ATAC-seq
H3K27ac
RNA-seq
MECOM LogFC 1.18
HA-PAX3
chr1:215,356,162-215,413,500
KCNK2 LogFC 1.39
G
TF Motif
FOS
JUN
HOXA13
POU5F1P1
SOX2
FOXA1
GATA3
MYOD1
OTX2
PAX3
RUNX1
PU.1
H
GATA3
Transcription Factor Motif Archetype
BAF Increase
BAF Decrease
Model Coefficients
I
ATAC-seq LogFC
SMARCA4 LogFC
J
HOXA9
MYOD1
RUNX1
Transcription Factor Motif Archetype
Model Coefficients
BAF Increase
BAF Decrease
K
5 PU.1 archetype motifs
3 PU.1 archetype motifs
1 PU.1 archetype motif
HA-SPI1
chr12:9,268,032-9,271,486
chr3:120,343,073-120,347,605
chr1:160,680,625-160,684,000
HA
SMARCA4
ATAC-seq
RNA-seq

Ectopic expression of TFs direct mSWI/SNF genomic targeting and activity. B. Immunoblots showing expression of HA-tagged TFs expressed in hMSCs from whole cell extracts. **B.** Expression (Log(1+RPKM)) of the TFs overexpressed in MSCs relative to their endogenous level of expression in control cells transduced with empty vector. **C.** Enrichment (RPKM) of HA-tagged TF, SMARCA4 and DNA accessibility (ATAC-Seq) at TF-Specific peaks (as labeled above each plot) in hMSCs expressing empty vector or TFs indicated on X-axis. **D.** Expression of genes closest to de novo mSWI/SNF peaks in MSCs expressing TF indicated on x-axis compared to their expression in control cells (EV). **E.** Occupancy (RPKM normalized) profiles of HA-tagged GATA3, SMARCA4 and DNA accessibility at 55,869 mSWI/SNF sites in MSCs expressing GATA3 or emoty vector control. Wildtype MSC-specific sites are on top and de novo GATA3-dependent peaks on the bottom. **F.** Example tracks of CUT&RUN profiles of HA-tagged GATA3 and PAX3, respectively, SMARCA4, DNA accessibility (ATAC-Seq) and gene expression (RNA-Seq) at the given locus. **G.** Homer motif enrichment (log_{10}p-value) of indicated TFs in peak clusters defined in Fig.1B. **H.** GLMnet motif enrichment analysis was performed to identify top TF motifs underlying SMARCA4 peaks that displayed gain of SMARCA4 enrichment (in red) and loss of enrichment (in blue) upon TF overexpression. **I.** Scatterplots displaying the correlation between change in SMARCA4 occupancy (x-axis) and DNA accessibility measured by ATAC-Seq (Y-axis) in MSCs expressing PU.1 compared to empty vector. Color key indicates PU.1 RPKM enrichment (top panel), number of PU.1-motifs (middle panel) and number of AP.1 motifs (lower panel). **J.** GLMnet motif enrichment analysis was performed to identify top TF motifs underlying SMARCA4 peaks that displayed gain of SMARCA4 enrichment (in red) and loss of enrichment (in blue) upon TF overexpression. **K.** Example tracks of CUT&RUN profile of HA-tagged PU.1, SMARCA4, DNA accessibility (ATAC-Seq) and gene expression (RNA-Seq) at loci containing 5, 3 and 1 PU.1 archetypical motifs, repectively, underlying the SMARCA4 peak shown in blue.

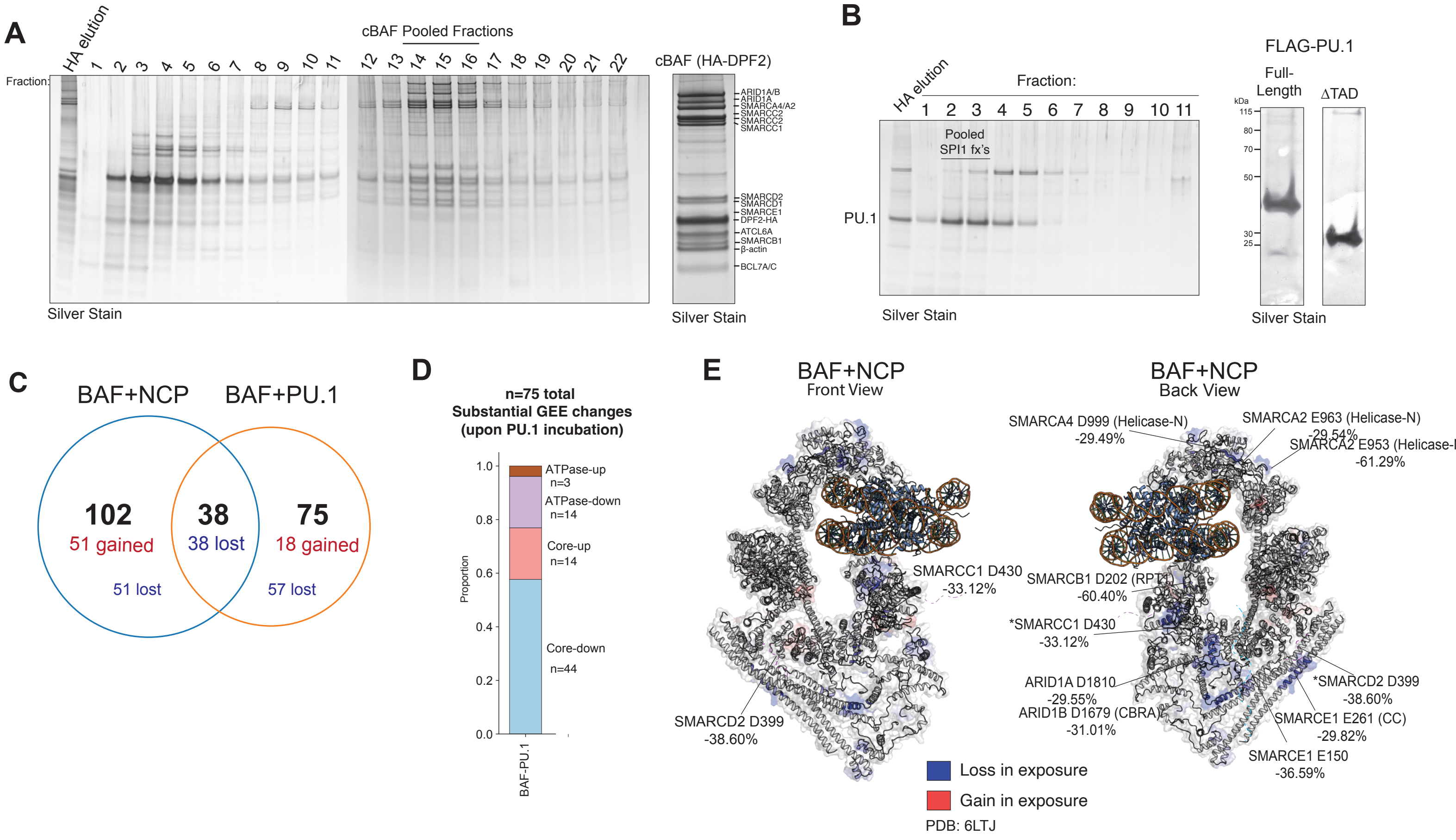

Figure S3. Protein footprinting experiments to identify the PU.1 interaction interface on the human cBAF complex. A. Purification of endogneous mammalian cBAF complexes from HEK-293T cells using HA affinity purification of HA-tagged DPF2 subunit, followed by density sedimentation. Silver stains for cBAF gradient fractions (left) and pooled fractions (Fx 14-15, right)**. B.** Silver stained SDS-PAGE showing purification of PU.1 using HA affinity purification followed by density gradient sedimentation for GEE experiments. **C.** Venn diagram showing overlap between peptides exhibiting changes in GEE labeling of solvent exposed residues upon incubation with NCP or PU.1. **D.** Distribution of cBAF peptides differentially labeled upon PU.1 interaction within ATPase or Core module subunits. **E.** cBAF peptides with substantial changes in GEE labeling upon NCP binding are mapped on 3D structure of cBAF-NCP cryo-EM structure (PDB: 6LTJ).

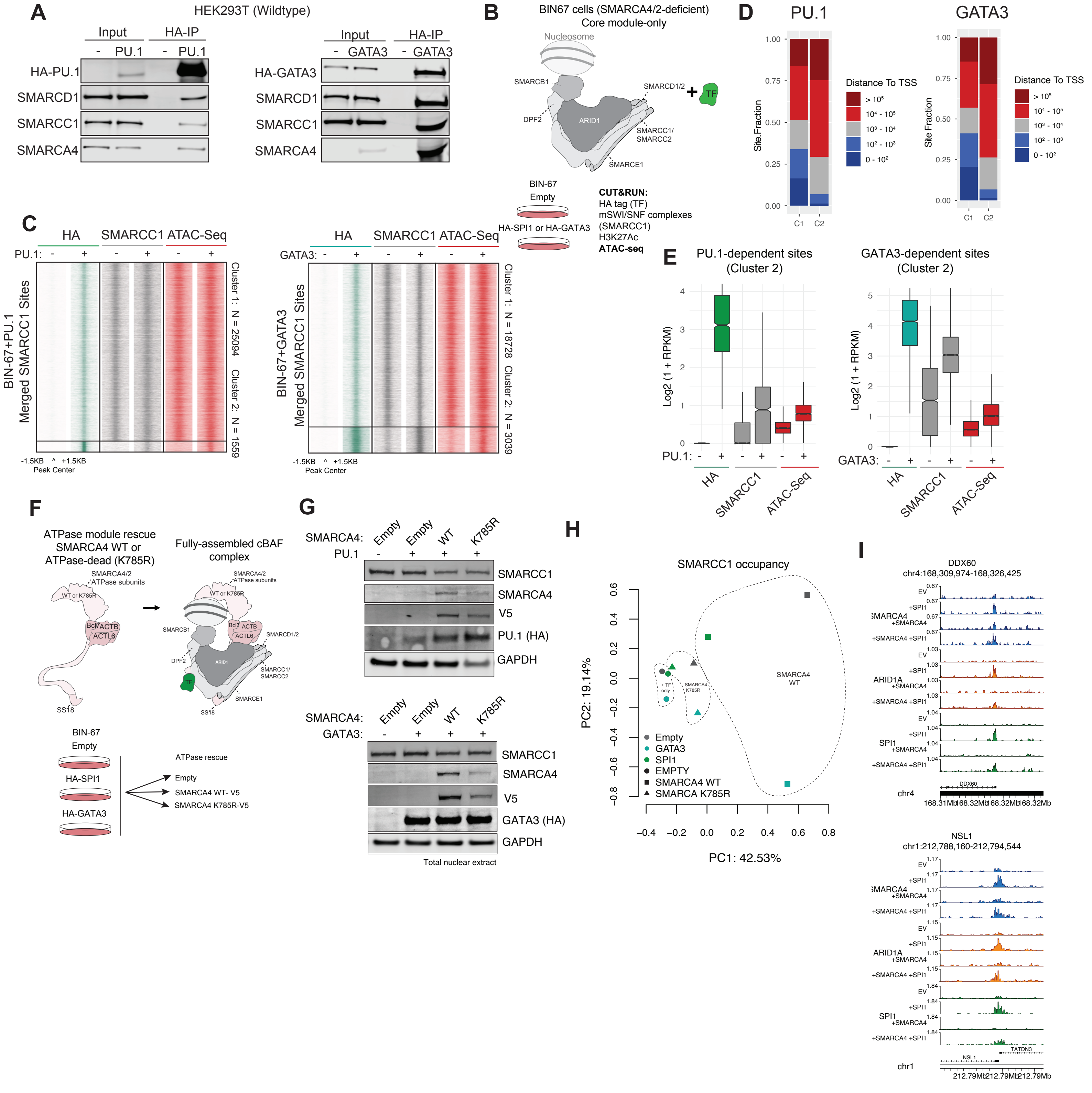

Figure S4. The cBAF core is sufficient for PU.1 and GATA3 TF interaction and mSWI/SNF chromatin targeting in cells. **A.**Immunoblots showing the eluates from immunoprecipitation of HA-tagged PU.1 and GATA3 expressed in wildtype HEK293T cells. **B.** HA-tagged PU.1 or GATA3 were expressed in SMARCA2/SMARCA4-deficient BIN67 SCCOHT cells and occupancy of mSWI/SNF core module subunits were probed using CUT&RUN. **C** Heatmaps displaying the occupancy profiles of HA-TF, SMARCC1 and DNA accessibility (ATAC-Seq) in BIN67 cells expressing HA-tagged PU.1 (left) or GATA3 (right) at all SMARCC1. Wildtype cell-specific loci (n= 25094 and 18726) and TF-dependent sites (n= 1559 and 3039) are shown in two clusters on top and bottom, respectively. **D.** Distance to TSS stacked bar graphs quantifying the occupancies of HA-tagged TF, SMARCC1 and DNA accessibility at TF-dependent sites in BIN67 cells expressing PU.1 or GATA3 from (C). **E.** Occupany of HA-tagged TF and SMARCC1, ATAC-seq over TF-dependent sites (Cluster 2). F. Schematic for experiments rescuing SMARCA4 WT or ATPase dead (K785R) relative to empty vector control in BIN-67 SSCCOHT cells. G. Expression of PU.1 or GATA3 TFs with or without concomitant rescue of SMARCA4 WT or ATPase-dead variants. H. PCA analysis of SMARCC1 occupancy across conditions indicated. I. Representative tracks at the DDX80 and NSL1 loci depicting TF-mediated redirection of the mSWI/SNF core module.

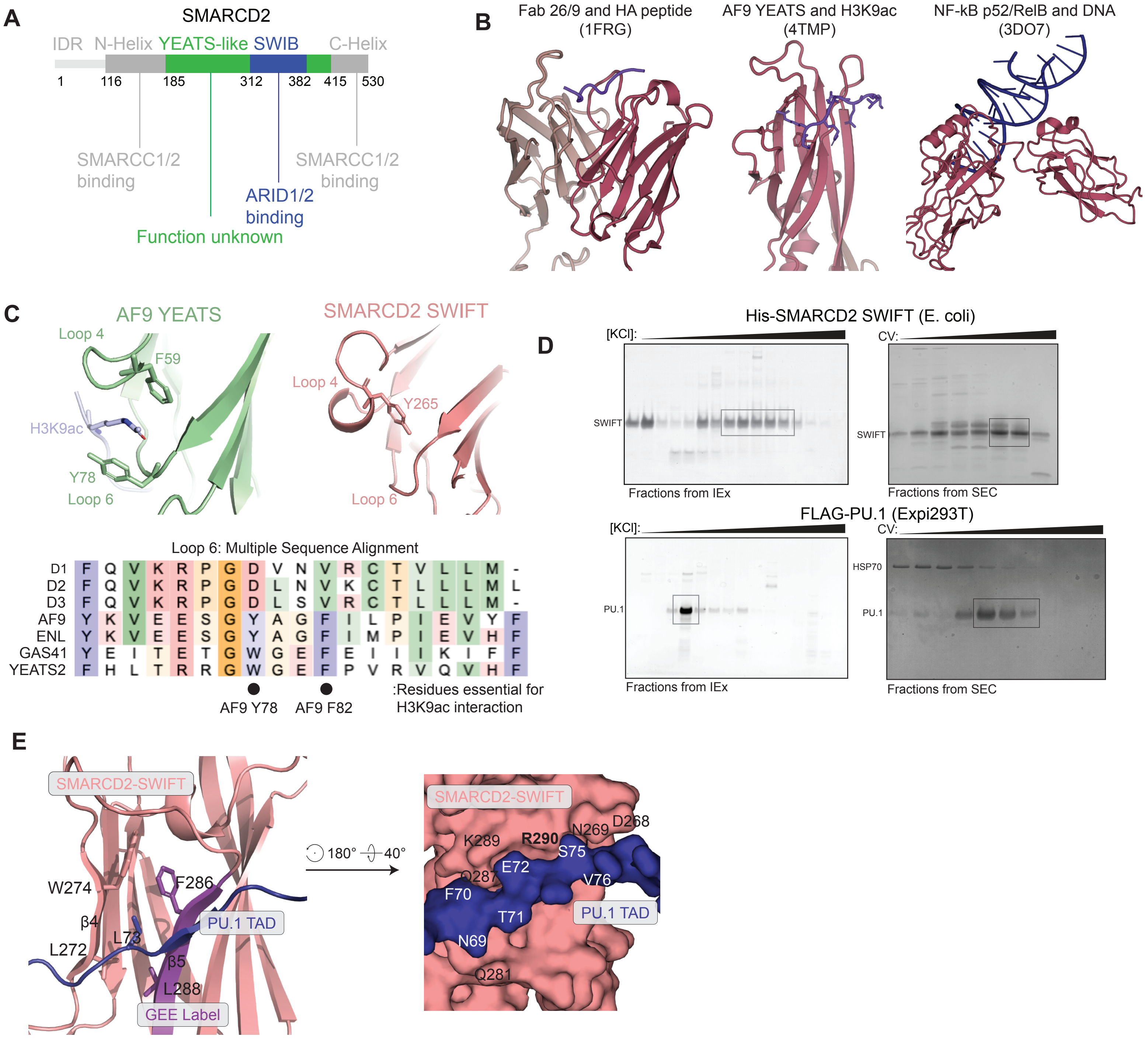

Figure S5. Characterization of the SMARCD Ig-fold-like SWIFT domain. A. Domain structure of SMARCD2 subunit of SWI/SNF and known structural functions and binding within mSWI/SNF complexes. **B.** Structural overview of immunoglobulin domains and their diverse interaction partners found in (from left to right) IgG with HA peptide, AF9 YEATS domain with H3K9ac peptide, NFkB bound to its cognate DNA. **C.** Structural comparison of AF9 H3K9ac-binding aromatic tunnel formed between loop 4 and loop 6. Absolutely essential F59 and Y78 residues engage in a ∏-∏-∏ interaction with H3K9ac group. Multiple sequence alignment of the loop 6 of human SMARCD1/2/3 SWIFT domain with YEATS domains from AF9, ENL, GAS41 and YEATS2. The essential resides (Y78 and F82) for H3K9ac interaction are absent from the SWIFT domain loop 6, suggesting a functional divergence. **D.** Purification of SWIFT domain from E. coli and PU.1 from Expi293F cells using anion exchange followed by size exclusion chromatography for in vitro binding studies. **E.** AlphaFold multimer modeling of SMARCD2 SWIFT domain with PU.1 showing a interaction between PU.1 L73 and hydrophobic pocket residues on SWIFT domain. Space filling model of interaction interface is shown on the right, displaying the proximity of electronegative and acidic residues to K289 and R290 basic residues.

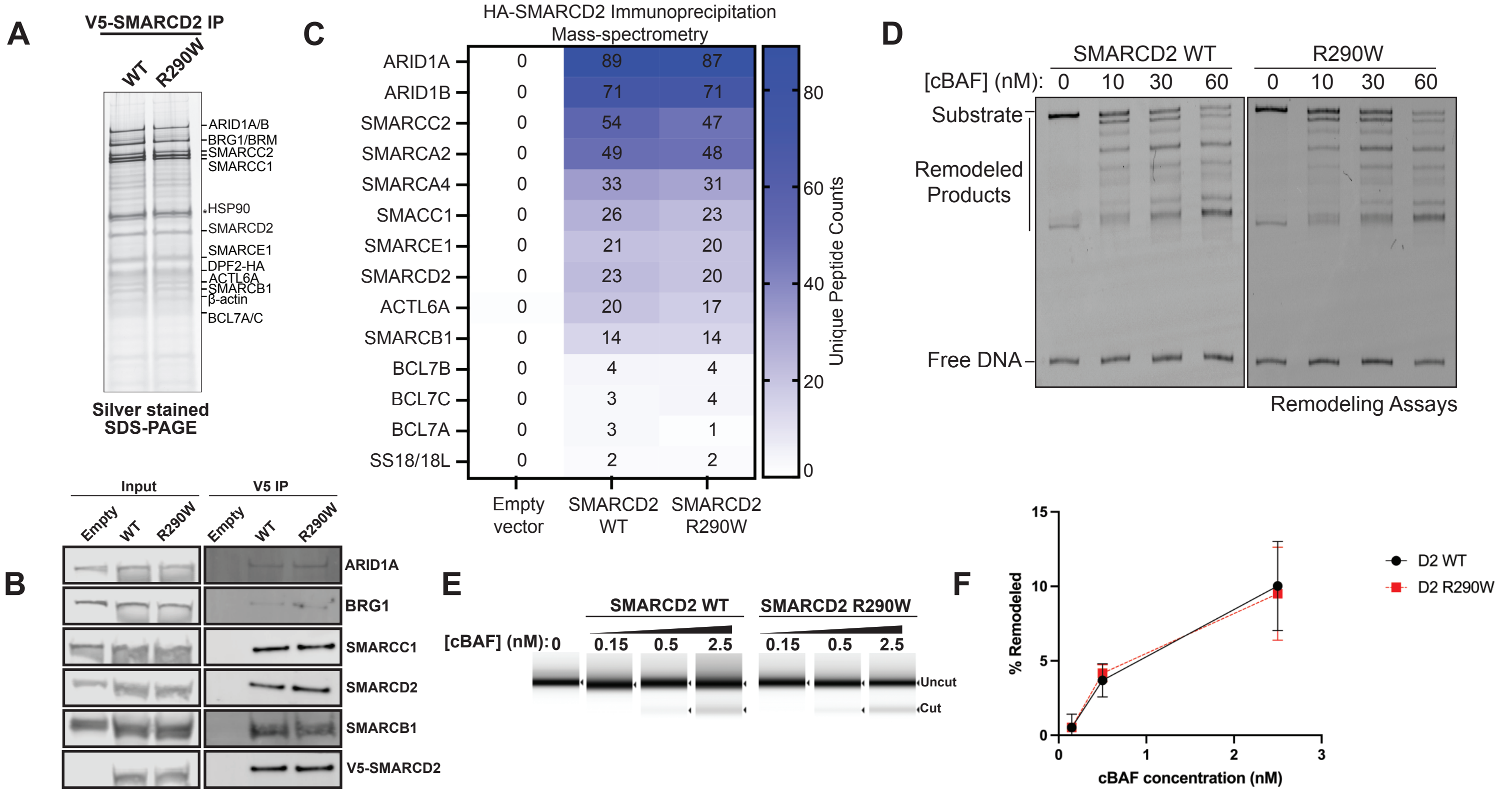

Figure S6. SMARCD2 containing the R290W SWIFT point mutation assembles into enzymatically active mSWI/SNF complexes. A. Silver stained SDS-PAGE showing the subunits of mSWI/SNF complexes co-purified with V5-tagged SMARCD2 from HEK-293T cells. **B.** Immunoblots of select mSWI/SNF complex subunits immunoprecipitated with V5-tagged SMARCD2 WT or R290W mutant. **C.** Proteomic mass spectrometric analysis of mSWI/SNF complex subunits purified with V5-tagged SMARCD2 WT or R290W mutant. **D.** Nucleosome sliding assays using 50 nM nucleosomes incubated with varying concentrations of mSWI/SNF containing SMARCD2 WT or R290W mutant for 30 min. Products were visualized using native PAGE gel. **E-F.** 5 nM nucleosomes were incubated with varying concentrations of mSWI/SNF complexes containing SMARCD2 WT or R290W mutant, ATP and DpnII restriction enzyme for 30 min. Remodeled products quantified by measuring DNA size distribution.

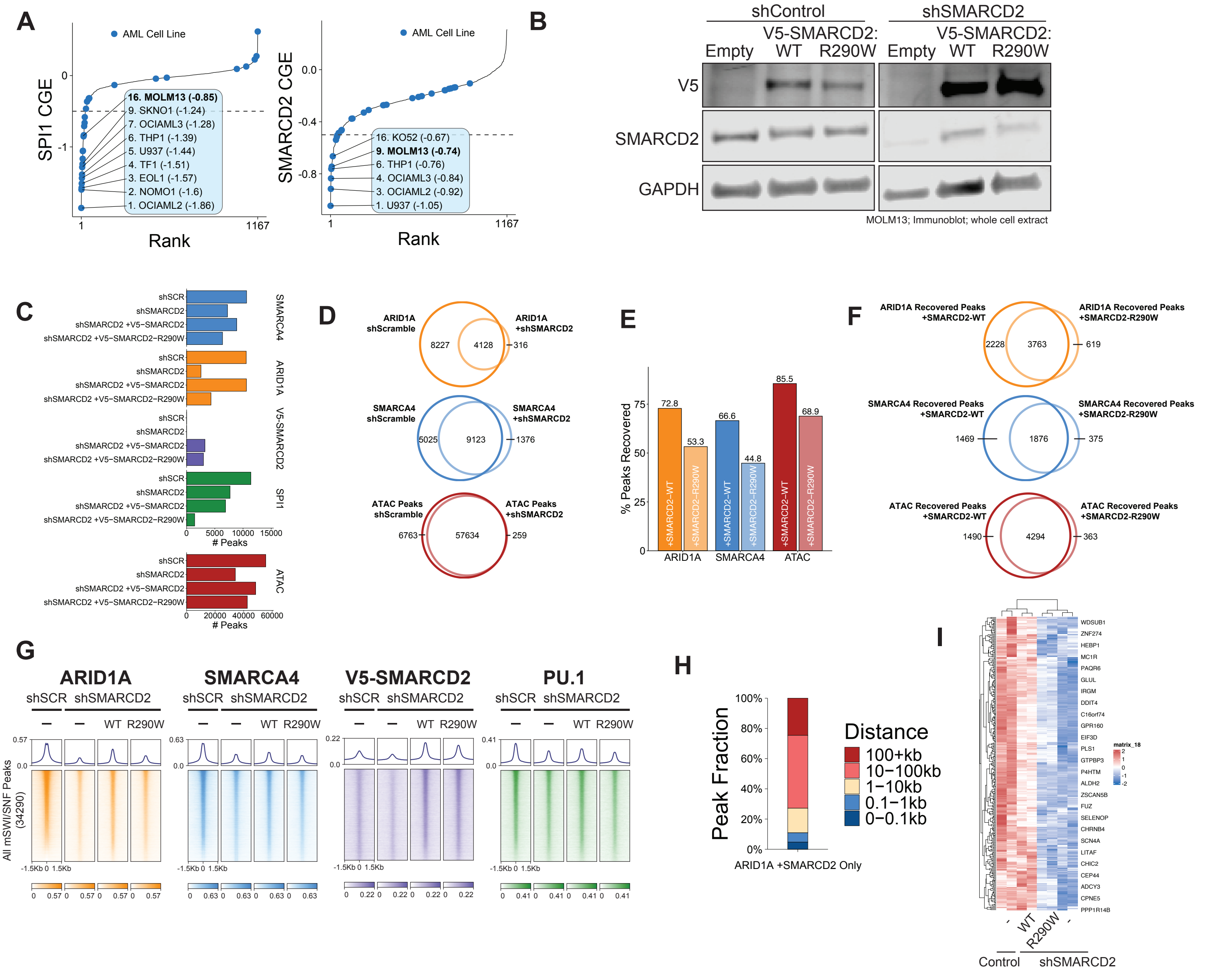

Figure S7. SWIFT-mediated interaction between cBAF and PU.1 is necessary for genomic targeting and activity of mSWI/SNF in MOLM-13 AML cells. (A) 1167 cell lines are ranked by their CRISPR dependency score (CRISPR gene effect, CGE) of PU.1 (left) or SMARCD2 (right). AML cell lines are marked in blue circles. **B>** Immunoblots of whole cell extracts from MOLM-13 cells expressing shRNA to target endogenous SMARCD2 or a non-targeting control, and rescued with a shRNA-resistant SMARCD2 WT or R290W transgene. **C.** Number of SMARCA4, ARID1A, V5-tagged SMARCD2 and PU.1 CUT&RUN peaks identified in SMARCD2 knockdown MOLM13 cells rescued with SMARCD2 WT or R290W transgenes. Number of ATAC-Seq peaks are shown below in red. **D.** Overlap between ARID1A, SMARCA4 and ATAC-Seq peaks in MOLM13 cell expressing shRNA against SMARCD2 or a non-targeting control. **E.** Percentage of overall ARID1A, SMARCA4 and ATAC-Seq peaks recovered by expression of shRNA-resistant SMARCD2 WT or R290W transgene. **F.** Overlap between the Peaks recovered upon re-expression of SMARCD2 WT or R290W point mutant. **G.** Heatmaps displaying the ARID1A, SMARCA4, V5-tagged SMARCD2 transgene and PU.1 occupancies in MOLM13 cells at 34,280 merged mSWI/SNF target sites. **H.** Stacked bar chart showing the distance from transcription start sites of the ARID1A peaks recovered by re-expression of SMARCD2 WT, but not R290W mutant. **I.** Heatmap showing the z-score normalized gene expression RPKM of selected known PU.1-target genes.

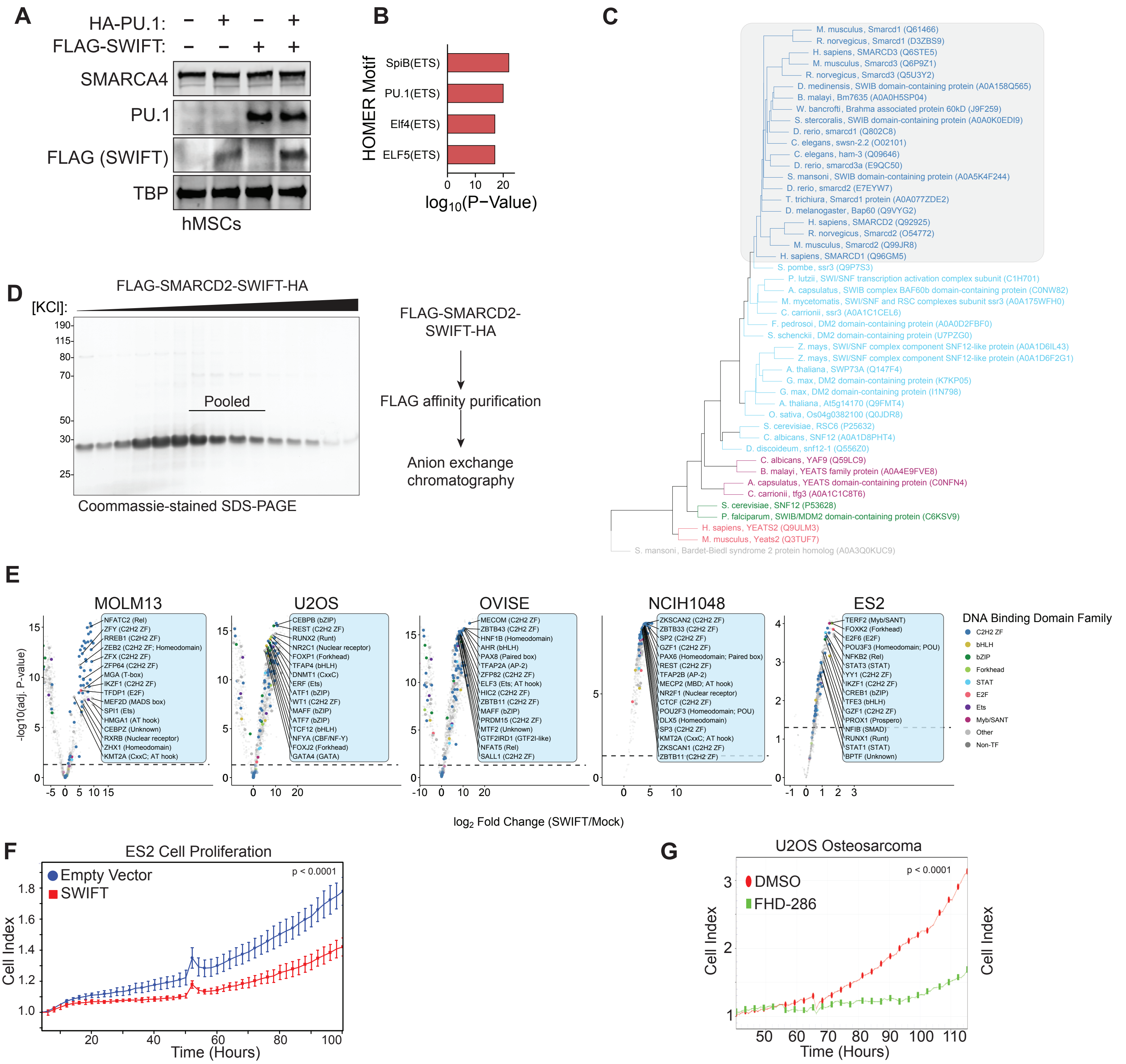

Figure S8. SWIFT is a transcription factor interaction platform for mSWI/SNF. A. Immunoblots of whole cell extract from hMSCs overexpressing PU.1 in the presence or absence of ectopic expression of FLAG-tagged SMARCD2 SWIFT domain. **B.** HOMER motif enrichment analysis of PU.1-dependent de novo SMARCA4 genomic target loci. **C.** Phylogenetic analysis of SWIFT domain primary sequence reveals its evolutionary conservation throughout the phylogenetic tree. **D.** Purification of N-terminally FLAG-tagged and C-terminally HA-tagged SMARCD2 SWIFT domain from mammalian Expi293F cells using FLAG affinity purification. **E.** Scatterplots displaying the enrichment of TFs bound by 200 nM SWIFT domain incubated with ammonium sulfate-extracted nuclear lysates from n=5 human cancer lines spanning diverse tissue types. **F.** Proliferation of U2OS cells overexpressing SWIFT domains, monitored by eSight. P-value $p<0.0001$ of difference in the slope of exponential growth for each cell line (n= 3 replicates) is indicated. **G.** Proliferation of U2OS cells treated with 100 nM FHD-286 or DMSO control (n= 2 replicates). P-value $p<0.0001$ of difference in the slope of exponential growth for each cell line is indicated.
